# VAMP3 and SNAP23 as Potential Targets for Preventing the Disturbed Flow-Accelerated Thrombus Formation

**DOI:** 10.3389/fcell.2020.576826

**Published:** 2020-11-05

**Authors:** Juan-Juan Zhu, Zhi-Tong Jiang, Chen Liu, Yi-Feng Xi, Jin Wang, Fang-Fang Yang, Wei-Juan Yao, Wei Pang, Li-Li Han, Yong-He Zhang, An-Qiang Sun, Jing Zhou

**Affiliations:** ^1^Department of Physiology and Pathophysiology, School of Basic Medical Sciences, Peking University, Beijing, China; ^2^Key Laboratory of Molecular Cardiovascular Sciences, Ministry of Education, Beijing, China; ^3^National Health Commission of the People's Republic of China Key Laboratory of Cardiovascular Molecular Biology and Regulatory Peptides, Peking University, Beijing, China; ^4^Department of Pharmacology, School of Basic Medical Science, Peking University, Beijing, China; ^5^Department of Clinical Laboratory, Peking University People's Hospital, Beijing, China; ^6^School of Biological Science and Medical Engineering, Beijing Advanced Innovation Center for Biomedical Engineering, Beihang University, Beijing, China

**Keywords:** thrombosis, VAMP3, endothelial cells, disturbed flow, SNAP23, VWF

## Abstract

Disturbed blood flow has been recognized to promote platelet aggregation and thrombosis via increasing accumulation of von Willebrand factor (VWF) at the arterial post-stenotic sites. The mechanism underlying the disturbed-flow regulated endothelial VWF production remains elusive. Here we described a mouse model, in which the left external carotid artery (LECA) is ligated to generate disturbed flow in the common carotid artery. Ligation of LECA increased VWF accumulation in the plasma. Carotid arterial thrombosis was induced by ferric chloride (FeCl_3_) application and the time to occlusion in the ligated vessels was reduced in comparison with the unligated vessels. *In vitro*, endothelial cells were subjected to oscillatory shear (OS, 0.5 ± 4 dynes/cm^2^) or pulsatile shear (PS, 12 ± 4 dynes/cm^2^). OS promoted VWF secretion as well as the cell conditioned media-induced platelet aggregation by regulating the intracellular localization of vesicle-associated membrane protein 3 (VAMP3) and synaptosomal-associated protein 23 (SNAP23). Disruption of vimentin intermediate filaments abolished the OS-induced translocation of SNAP23 to the cell membrane. Knockdown of VAMP3 and SNAP23 reduced the endothelial secretion of VWF. Systemic inhibition of VAMP3 and SNAP23 by treatment of mice with rapamycin significantly ameliorated the FeCl_3_-induced thrombogenesis, whereas intraluminal overexpression of VAMP3 and SNAP23 aggravated it. Our findings demonstrate VAMP3 and SNAP23 as potential targets for preventing the disturbed flow-accelerated thrombus formation.

## Introduction

Arterial thrombosis is thought to initiate with events such as rupture or erosion of atherosclerotic plaques, vessel damage, and dysfunction of vascular endothelium. In advanced atherosclerosis, plaques become stenotic and can cause progressive obstruction of the arterial lumen, and correspondingly, the local arterial geometries change to result in alteration of the hemodynamic microenvironment (Hyun et al., 2000). Computational simulation of stenotic flow has indicated that the wall shear rate peak sharply just upstream of the apex of the stenosis, with sustained high shear conditions within the stenosis (Hathcock, 2006). The flow pattern upstream of and within the stenosis is typically characterized as laminar or pulsatile. Fluid layers may separate from each other as it decelerates downstream of the stenosis to form stagnation zones (with near-zero wall shear stress) and areas of recirculation, resulting in a transition of laminar to disturbed flow (Hyun et al., 2000; Hathcock, 2006). Disturbed flow with unorganized, chaotic flow regime has been recognized to influence vascular endothelial behavior and therefore favors platelet aggregation by increasing accumulation of von Willebrand factor (VWF) at post-stenotic sites (Westein et al., 2013). Although flow disturbance has a well-defined role in impairing endothelial function, the mechanisms by which disturbed blood flow affects thrombotic process in particular the endothelial secretion of VWF remain poorly understood. Moreover, appropriate animal models to study the flow-regulated thrombosis formation are ill-defined.

Thrombogenic component VWF is a multimeric glycoprotein, stored in secretory vesicles called Weibel-Palade bodies (WPBs) in endothelial cells, α-granules in megakaryocytes and platelets (Wagner et al., 1982; Sporn et al., 1985). Plasma VWF is produced mainly by endothelial cells, with a minor contribution from megakaryocytes or platelets (Valentijn and Eikenboom, 2013). VWF secretion can occur from both apical and basolateral sides of endothelial cells via constitutive, basal, and regulated secretory pathways, the latter two are dependent on WPB exocytosis (Lopes da Silva and Cutler, 2016). Thrombogenic stimuli such as fluid turbulence may trigger a regulated rapid exocytosis of endothelial WPBs and a release of VWF, which interact with platelet glycoprotein Ib-IX-V receptor, integrin α_IIb_β_3_ and other components of the exposed sub-endothelium to initiate the primary thrombus formation (Mustard et al., 1962, 1966). WPB exocytosis is a multistep process that includes tethering, docking, priming, and membrane fusion (Burgoyne and Morgan, 2003). Regulation of WPB exocytosis involves multiple layers of cellular machineries with the cooperation of vesicle secretion-related proteins such as Rab proteins (Rab27a, Rab27b, Rab3A, Rab15b) (Zografou et al., 2012), Rab effector proteins (MyRIP, granuphilin/Slp4a, Slp2a, Slp3, Munc13-4, Munc18c, Noc2) (Bierings et al., 2012; Zografou et al., 2012; Nightingale and Cutler, 2013), vesicle transport-associated proteins SNAREs (soluble N-ethylmaleimide-sensitive factor attachment protein receptors) (Valentijn et al., 2011), and the small GTPase RalA (de Leeuw et al., 2001). SNARE complexes are the core components of the exocytosis machinery for WPB. Studies have shown that the SNARE complexes that consist of the WPB exocytosis machinery contain vesicle-associated membrane protein (VAMP) 3, VAMP8, syntaxin4 and synaptosomal-associated protein 23 (SNAP23) (Predescu et al., 2005; Pulido et al., 2011; Zhu et al., 2015). SNAP23 is the only SNAP homolog that localizes on the endothelial cell membrane and regulates endothelial cell exocytosis, deficiency of SNAP23 can impair endothelial exocytosis. SNAP23 has been reported to associate with vimentin intermediate filaments in fibroblasts, where vimentin filaments serve as a reservoir for SNAP23 (Faigle et al., 2000). VAMP3 are present on WPB, forming stable complexes with SNAP23, the plasma membrane-associated SNAREs in endothelial cells to allow fusion of WPB with the plasma membrane (Pulido et al., 2011). In previous study we found that disturbed blood flow upregulates the expressions of VAMP3 and SNAP23 and enhances their interaction in vascular endothelia. How important the hemodynamically drove regulation on VAMP3 and SNAP23 is in promoting thrombus formation remains unclear.

In the present study, we developed an animal model to investigate the disturbed flow-accelerated thrombosis in the murine carotid artery *in vivo*. In addition, we studied the influences of shear stress with distinct flow patterns on VWF secretion as well as the underlying regulatory mechanism in cultured endothelial cells by using parallel-plate flow apparatus. Our data show that, both *in vivo* and *in vitro*, the disturbed fluid shear stress aggravates VWF secretion and local platelet aggregation in a VAMP3/SNAP23 dependent manner. We report for the first time that vimentin associates with SNAP23 to modulate the oscillatory shear stress-induced translocation of SNAP23 to the cell membrane. This work highlights a critical role for the mechano-sensitive SNAREs in converting the hemodynamic microenvironment into prothrombotic responses and suggests VAMP3 and SNAP23 as potential targets for preventing the disturbed flow-accelerated thrombus formation.

## Materials and Methods

### Cell Culture

Human umbilical vein endothelial cells (HUVECs) were cultured in medium 199 (M-199) supplemented with 10% fetal bovine serum (FBS), 4 μg/mL of β-Endothelial Cell Growth Factor (Sigma, Burlington, MA, USA), 1% penicillin/streptomycin (Harvey-bio, Beijing, China), at 37°C with 5% CO_2_. Cells at passages 5–7 were used for all *in vitro* experiments. Human umbilical vein endothelial cells (HUVECs) were obtained from umbilical cords from healthy patients after full-term deliveries. Umbilical cords were obtained with the agreement of the patients and approved by the Peking University People's Hospital Medical Ethics Committee (2015PHB024).

### Parallel-Plate Flow Apparatus

The monocultured HUVECs seeded on glass slides pre-coated with collagen I (50 μg/mL) were subjected to shear stress in a parallel-plate flow chamber. Channel in the chamber was created by a silicon gasket with dimensions of 2.496 cm in width (*w*), 5.725 cm in length, and 0.025 cm in height (*h*). The system was kept in a constant-temperature controlled enclosure, with pH maintained at 7.4 by continuous gassing with a humidified mixture of 5% CO_2_, 20% O_2_, and 75% N_2_. The shear stress (τ) generated on the cells seeded on the membrane was estimated as 6*QA*μ/*wh*^2^, where *QA* is flow rate and μ is perfusate viscosity. The flow with pulsatile shear (PS, 12 ± 4 dynes/cm^2^) or oscillatory shear (OS, 0.5 ± 4 dynes/cm^2^) is composed of a low level of mean flow with shear stress at 12 or 0.5 dynes/cm^2^ supplied by a hydrostatic flow system to provide the basal nutrient and oxygen delivery, and the superimposition of a sinusoidal oscillation using a piston pump with a frequency of 1 Hz and a peak-to-peak amplitude of ± 4 dynes/cm^2^. Cells were exposed to either PS or OS for the indicated time periods. In some experiments, the conditioned media collected from sheared cells were used to study VWF accumulation and its influences on platelet aggregation.

### Antibodies, Reagents, and Adenovirus

Rabbit polyclonal antibody (pAb) against VAMP3, rabbit pAb against E-selectin, rabbit pAb against ICAM-1 were purchased from Proteintech (Chicago, Illinois, USA). Rabbit pAb against SNAP23 was from Abcam (Cambridge, United Kingdom). Mouse pAb against CD31 and mouse mAb against VCAM1 were from Santa Cruz Biotechnology (Dallas, TX, USA). Rabbit pAb against VWF was from Dako (Agilent, Beijing, China). Rabbit pAb against GAPDH was from Easybio (Beijing, China). Mouse monoclonal antibody against Vimentin was from Bioss (Beijing, China). Rapamycin was from Alexis. Recombinant adenovirus expressing GFP-fused full-length human SNAP23 (Ad-SNAP23) or VAMP3 (Ad-VAMP3) and the control adenovirus expressing GFP (Ad-GFP) only were obtained from Vigene Biosciences (Jinan, Sandong, China). SiRNAs for knocking down VAMP3 or SNAP23 and the control siRNAs were from GenePharma (soochow, Jiangshu, China).

### Infection and Transient Transfection

For loss-of-function study of VAMP3 and SNAP23, cells at 80% confluence were transfected with siRNAs specific for VAMP3 (#1, 5′-CACUGUAAUCACCUAAAUAAATT-3′, #2, 5′-CCCAAAUAUGAAGAUAAACUATT-3′, GenePharma), SNAP23 (5′-CUUUGAGUCUGGCAAGGCUTT-3′, GenePharma), or the scrambled siRNA control using lipofectamine 2000 transfection agent (Thermo Fisher Scientific, Waltham, MA, USA) according to the manufacturer's instructions. The efficiency of siRNA-mediated knockdown on SNAP23 and VAMP3 was tested (Zhu et al., 2017). For gain-of-function study of VAMP3 and SNAP23, cells were infected with Ad-VAMP3, Ad-SNAP23 or Ad-GFP.

### Western Blot Assay and Sodium Dodecyl Sulfate (SDS)-Polyacrylamide Gel Electrophoresis

Cells were lysed in RIPA lysis buffer: 50 mM Tris (pH 7.4), 1% NP-40, 0.5% deoxycholate, 0.1% SDS, 150 mM NaCl, 5 mM EDTA, 50 mM NaF, protease inhibitor cocktail tablets (Roche, Basel, Switzerland). Equal amounts of proteins were separated on SDS-polyacrylamide gel electrophoresis, transferred to nitrocellulose membranes. Non-specific binding was blocked in 5% skimmed milk in Tris-buffered saline (TBS) containing 0.1% Tween 20, and then the membranes were incubated with specific primary antibodies overnight at 4°C, followed by their detection using donkey anti-rabbit/-mouse/-goat IgG (H&L) antibody IRDye 800/700 Conjugated (Rockland, Philadelphia, Pennsylvania, USA). Visualization was performed with an Odyssey infrared imaging system (LI-COR Biosciences).

### Western Blot Assay and SDS-Agarose Gel Electrophoresis

Plasma and the conditioned media collected from sheared cells were diluted in sample buffer [10 mM Tris, 1 mM Na_2_EDTA, 2% (w/v) SDS, 8 M Urea, 2 mg/mL bromophenol blue]. The ratio of plasma or conditioned media to sample buffer is 1:10 and 1:5, respectively. The dilutions were incubated at 60°C for 30 min. Stack gel [0.8% agarose (w/v), 70 mM Tris, 4 mM EDTA, 0.4% SDS (W/V), pH 6.8] and separating gel [2% agarose (w/v), 0.1 M Tris, 0.1 M glycine, 0.4% (w/v) SDS, pH 8.8] were prepared. Equal amounts of diluted samples were loaded into the wells. The gel was subsequently soaked in running buffer [50 mM Tris, 384 mM glycine, 0.1% SDS (W/V) pH 8.3] and was subjected to electrophoretic migration process at a constant current density of 10 mA. After electrophoresis, the gel was soaked in transfer buffer (48 mM Tris, 39 mM glycine, 20% methanol, 0.037% SDS, pH 9.2) coated with 1 mM 2-mercaptoethanol (2-ME), and was then equilibrated for 30 min at room temperature. Following washing with transfer buffer (without 2-ME) for 15 min, the proteins in the gel were transferred at 4°C under a constant voltage of 30 V for 16 h to a 0.2-μm pore nitrocellulose membrane. The membrane was then incubated with polyclonal rabbit anti-VWF primary antibody at 4°C overnight. After washing with TBST buffer (60.57 g/L Tris Base, 87.75 g/L NaCl, 5 mL/L Tween 20, pH 7.4), the membrane was incubated with donkey anti-rabbit IgG antibody IRDye 800/700 Conjugated protected from light for 1 h at room temperature. Visualization was performed with an Odyssey infrared imaging system.

### Immunofluorescence

Tissues were fixed in 4% paraformaldehyde (PFA) and were embedded in 30% sucrose solution before being frozen in TissueTek cutting medium (Sakura Finetek). Six micrometer sections were processed for immunofluorescent analyses. For *en face* preparation of mouse tissues, the common carotid arteries were harvested and post-fixed in this 4% PFA for no more than 1 day. Tissues were first washed with phosphate-buffered saline (PBS) buffer and adventitia was removed carefully. Aortas were then longitudinally dissected with microdissecting scissors and pinned flatly on a black wax dissection pan. The luminal surfaces of the common carotid artery were immediately blocked with 3% bovine serum albumin (BSA) (w/v) for 1 h. For immunostaining of attached cells, the cells were fixed in 4% PFA for 15 min and were permeabilized with 0.25% Triton X-100 in PBS for 10 min. Nonspecific binding was blocked by 3% BSA in PBS. The sections/tissues/cells were probed with primary antibodies (CD31, VCAM1, ICAM1, VWF, SNAP23, VAMP3, VE-cadherin, Vimentin), washed and then probed with secondary antibodies including Alexa Fluor 488-conjugated donkey/goat anti-rabbit/-mouse IgG (1:500, Thermo Fisher Scientific) or Alexa Fluor 555-conjugated donkey anti-rabbit/-goat IgG (1:500, Thermo Fisher Scientific). Nuclei were counterstained with Hoechst 33342. After mounting, the slips were visualized by fluorescence microscopy (Leica DMI6000B; Leica TCS SP8, Wetzlar, Germany). To quantify co-localization, Pearson's correlation coefficient was calculated by ImageJ Coloc 2 Fiji's plugin for colocalization analysis.

### Proximity Ligation Assay (PLA)

Duolink *in situ* PLA was performed according to the protocol from the manufacturer (Sigma-Aldrich). In brief, HUVECs were sheared by PS or OS for 1 h, and then fixed with 4% PFA for 10 min at room temperature, washed with clod PBS three times and permeabilized with 0.5% Saponin for 10 min at room temperature. Unspecific binding sites for antibodies were blocked with blocking solution, the sample were incubated in a heated chamber for 1 h at 37°C. Next, the samples were incubated with primary antibodies solution of SNAP23 (1:100) and Vimentin (1:100) overnight at 4°C. Wash the samples in wash buffer A (0.01 mol/L Tris, 0.15 mol/L NaCl, and 0.05% Tween 20) at room temperature. Secondary antibodies conjugated with oligonucleotides (PLA probe MINUS and PLA probe PLUS) were added into the reaction and the mixtures were incubated in a pre-heated humidity chamber for 1 h at 37°C. Tap off the PLA probe solution from the samples. Samples were washed with wash buffer A and incubated with ligation solution for 30 min at 37°C. The Ligation solution consists of two oligonucleotides (illustrated as red bands) and ligase. Samples were then washed with buffer A and were incubated with amplification solution for 100 min at 37°C. The Amplification solution consists of nucleotides, fluorescently labeled oligonucleotides, and polymerase. The oligonucleotide arm of one of the PLA probes acts as a primer for a rolling-circle amplification (RCA) reaction using the ligated circle as a template, generating a concatemeric (repeated sequence) product. Tap off the amplification solution from the samples. Wash the samples in wash buffer B (0.2 mol/L Tris and 0.1 mol/L NaCl) at room temperature. Nuclei were counterstained with Hoechst 33342. After mounting, the samples were visualized by fluorescence microscopy (Leica TCS SP8). The ratio of fluorescence signal to cell number in each visual field was counted.

### VWF Level Was Quantitatively Detected by Enzyme Linked Immunosorbent Assay (ELISA)

HUVECs subjected to PS or OS for indicated hours were rinsed and incubated in serum-free M199 medium supplemented with 1% BSA for 1 h at 37°C. The conditioned media were then collected and the remaining cells were lysed with RIPA lysis buffer. Cell lysates were collected to determine total VWF levels. Relative amounts of VWF were determined by ELISA. Ninety-six-well plates were coated with the anti-VWF antibody (1:000) and were then blocked before adding the conditioned media. Serially diluted human plasma served as standards. The plates were washed, and the horseradish peroxidase (HRP)-conjugated anti-VWF antibody (1:2,000) was added. After further rounds of washing, the plates were developed with o-phenylenediamine dihydrochloride and hydrogen peroxide in citrate phosphate buffer. Absorbance was analyzed at 490 nm. Basal and the stimulated release are presented as a percentage of the total VWF present in the cells.

### Co-immunoprecipitation (Co-IP)

HUVECs were trypsinized and lysed with lysis buffer containing pH 7.4, 50 mM Tris-HCl, 100 mM NaCl, 1% NP-40, 1 mM EDTA, 1 mM PMSF, 1 mM NaVO4 and a protease inhibitor cocktail (Roche). Protein concentration of the lysate was adjusted to be 2–3 μg/μl. Five-hundred microliter of cell lysates were precleared with 1 μg of control IgG and 30 μl of protein A/G plus-agarose beads for 2 h rotation at 4°C. Five micro grams of SNAP23 antibody or corresponding IgG were added into the precleared cell lysates. After incubation and rotation at 4°C overnight, the immune complexes were pulled down with 30 μl of protein A/G plus-agarose beads and were washed with the lysis buffer. Sixty microliter of 2 × SDS buffer was added into each sample, which was then subjected to Western blot analysis.

### Platelet Aggregation Assay

Blood samples were obtained with the agreement and approved by the Peking University People's Hospital Medical Ethics Committee (2018PHB210-01) from healthy volunteers without hematological history, platelet or coagulation dysfunction, or taking drugs that may influence hematological function. Platelet-rich plasma (PRP) were obtained from anticoagulated whole blood by centrifugation at 800 rpm for 10 min at room temperature. PRP on the top were transferred to a clean Eppendorf tube. Platelet-poor plasma (PPP) were obtained from re-centrifuging the remaining blood samples at 3,000 rpm for 10 min at room temperature. PRP with the same volume were mixed with the conditioned media derived from sheared HUVECs or saline solution. PPP were mixed with saline solution. Platelet aggregation was triggered by the addition of adenosine diphosphate (ADP, 0.2 μM) (Ya et al., 2019; Chaudhary et al., 2020) and were monitored and recorded using an aggregometer (Chrono-Log, Model Number 700, U.S.A).

### Ligation of the LECA

All animal studies were performed in accordance with the approved protocol of the Animal Care and Use Committee of Peking University and approved by the Ethics Committee of Peking University Health Science Center (LA2018160). Anesthetization and euthanasia were performed by intraperitoneal injection of sodium pentobarbital (50 and 150 mg/kg, respectively). C57BL/6 wild-type mice (8–12 weeks old, 18–25 g) were anesthetized and were subjected to ligation of the left external carotid artery (LECA) with 6-0 silk suture, while other branches remained intact. After surgery, some mice were injected intraperitoneally every other day with rapamycin (1 mg/kg body weight) or with control reagent DMSO. Some mice received a locally intraluminal incubation of 50 μl per mouse of Ad-SNAP23 and Ad-VAMP3 viruses [a titer of 1 × 10^9^ plaque-forming units (pfu)/mL] or control virus for 30 min. Briefly, the left common carotid artery was exposed and the heart proximal end of the artery was clamped temporarily. The indicated adenoviruses were injected into the common carotid artery through the external carotid with an insulin needle, and then the LECA was immediately ligated. Clamp was removed after 30 min of incubation. Mice were kept in specific pathogen-free cages, 12 h light-dark cycle, controlled temperature and humidity, and had water and food *ad libitum*. One day or 1 week after ligation, the mice were sacrificed and fixed for 5 min by perfusion through left cardiac ventricle with 4% PFA in PBS buffer under physiological pressure. Carotid arteries were harvested and subjected to histology and immunostaining analyses of the vessels.

### High-Resolution Ultrasound Measurements

C57BL/6 mice were placed on a heat mat and were anesthetized. Hairs on the neck were removed using depilatory cream. Mice were subjected to ligation of the LECA or sham operation. After an indicated duration post-surgery, the mice were examined by using a Vevo 770 high-resolution ultrasound imaging system (FUJIFILM VisualSonics, Toronto, ON, Canada). The system was equipped with RMV-707B microimaging ultrasound system with a 30-MHz mouse probe (Visualsonics). Heart rate, temperature, and respirations were continuously monitored. Pulse wave doppler mode was used at the inlet, midpoint, and outlet of the common carotid arteries for measuring flow velocity.

### FeCl_3_-Induced Mouse Thrombosis Model

C57BL/6 mice were anesthetized and the left common carotid arteries (LCA) were isolated. A doppler flow probe (model 0.5 VB, Transonic Systems Inc, Ithaca, NY) was applied around the common carotid artery. The probe was connected to a flowmeter (transonic perivascular flow module, TS420). Blood flow in the vessel was monitored continuously. A section of a least 5 mm of the artery was isolated and 3 × 6 mm^2^ parafilm was placed below the artery. Two or three drops of saline were applied to the vessel to avoid drying up. A piece of filter paper saturated with 20% (w/v) FeCl_3_ solution was placed on the artery and incubated for 3 min (Liang et al., 2015b; Li et al., 2017). After incubation the artery was rinsed with saline. Thrombus formation was assessed by monitoring blood flow immediately after FeCl_3_ application and for up to 40 min, if the blood flow did not stop after 40 min, the time of thrombus formation was recorded as 40 min (Allende et al., 2017). The time to occlusion was calculated as the time required for blood flow through the carotid artery was monitored until vessel occlusion reached 95% (Liang et al., 2015a).

### Real-Time Observation of Carotid Artery Thrombosis

C57BL/6 mice were anesthetized and were subjected to ligation of the LECA. The right jugular vein was exposed, and 150 μL of 0.5 mg/mL rhodamine 6G solution (labeling platelets) were injected through the vein using an insulin syringe with 30 G needle (2–3 mm tip of the needle was bent to a 90° angle with a needle holder) (Li et al., 2016). The injection site was clamped using a hemostat and was then ligated with 6-0 silk suture. The left common carotid artery was isolated and was then subjected to 20% FeCl_3_-induced thrombosis. Thrombus formation was immediately observed and recorded using a fluorescence microscope in real-time.

### Magnetic Resonance Image (MRI) Scan, Three-Dimensional Reconstruction (3D) and Blood Flow Simulation

To obtain the *in vivo* geometries of carotid arteries, one mouse was selected for MRI scanning (192 × 192 image resolution, 1 mm slice thickness, 7.0 T Bruker BioSpec 70/20 USR) before and 1 week after ligation in the National Center for Nanoscience and Technology of China. MRI scan images were segmented, 3D reconstructed and smoothed using Mimics (Materialize Interactive Medical Image Control System v15.0, Materialize, Ann Arbor, MI, USA) and Geomagic (Geomagic Studio, v12, Geomagic, USA). Pulsatile velocity selected from the ultrasound measurements of the two groups (before and 1 week after ligation) were used as the inlet boundary condition and outflow was used as the outlets condition with split ratio of 0.683:0.317 (IC:EC). Mesh generation and flow simulation were conducted using commercial software ANSYS ICEM CFD (ANSYS Inc., Canonsburg, PA). In the simulation process, the vessel wall was assumed as rigid. Blood was assumed as Newtonian, homogeneous and incompressible fluid (Sakalihasan et al., 2005; Li and Kleinstreuer, 2006). Numerical simulations were based on the Navier-Stokes equation and the continuity equation:

(1) $$\begin{array}{r} \rho \left[\frac{\partial \vec{u}}{\partial t}+\left(\vec{u}\cdot \nabla \right)\vec{u}\right]+\nabla p-\nabla \cdot \tau =0 \end{array}$$

(2) $$\begin{array}{r} \nabla \cdot \vec{u}=0 \end{array}$$

Where μ is the velocity vector, ρ = 1,060 Kg/m^3^ is blood density, μ = 0.0035 Pas is the dynamics viscosity (Feintuch et al., 2007; Nemeth et al., 2009), p is the pressure and τ is the stress tensor.

The time average wall shear stress (TAWSS) is the average of the wall shear stress in one cardiac cycle:

(3) $$\begin{array}{r} TAWSS=\frac{1}{T}\int_{0}^{T}|WSS\left(s,t\right){|}_{▪}dt \end{array}$$

Where T is the cardiac cycle time.

The Oscillation Shear Index (OSI) is a measure of the change in the direction of shear stress (He and Ku, 1996), which is often used to describe how even in time does the WSS positively and negatively change. The high OSI usually accompanies with low and oscillated flow condition:

(4) $$\begin{array}{r} OSI=0.5\left[1-\left(\frac{\left|\frac{1}{T}\int_{0}^{T}WSS\left(s,t\right)▪dt\right|}{\frac{1}{T}\int_{0}^{T}WSS\left(s,t\right)▪dt}\right)\right] \end{array}$$

Where T is the cardiac cycle, and WSS is wall shear stress vector.

Relative residence time (RRT) is a parameter to evaluate the resident time of the blood flow and indicate regions suffering from either low WSS or high OSI. It is inversely proportional to the magnitude of the TAWSS vector and has obvious connections to the biological mechanisms of atherosclerosis (Lee et al., 2009):

(5) $$\begin{array}{r} RRT=\frac{1}{\left(1-2▪OSI\right)▪TAWSS} \end{array}$$

### Statistical Analysis

Data are expressed as mean ± standard error of the mean (SEM) from at least three independent experiments. Statistical analysis was performed by unpaired *t*-test for two groups of data and by one-way ANOVA for multiple comparisons. Statistical significance among multiple groups was determined by *post hoc* analysis (Tukey honestly significant difference test). Values of *P* < 0.05 were considered to be statistically significant.

## Results

### Ligation of LECA Causes Low and Disturbed Blood Flow in the Common Carotid Artery

**Figure 1:**
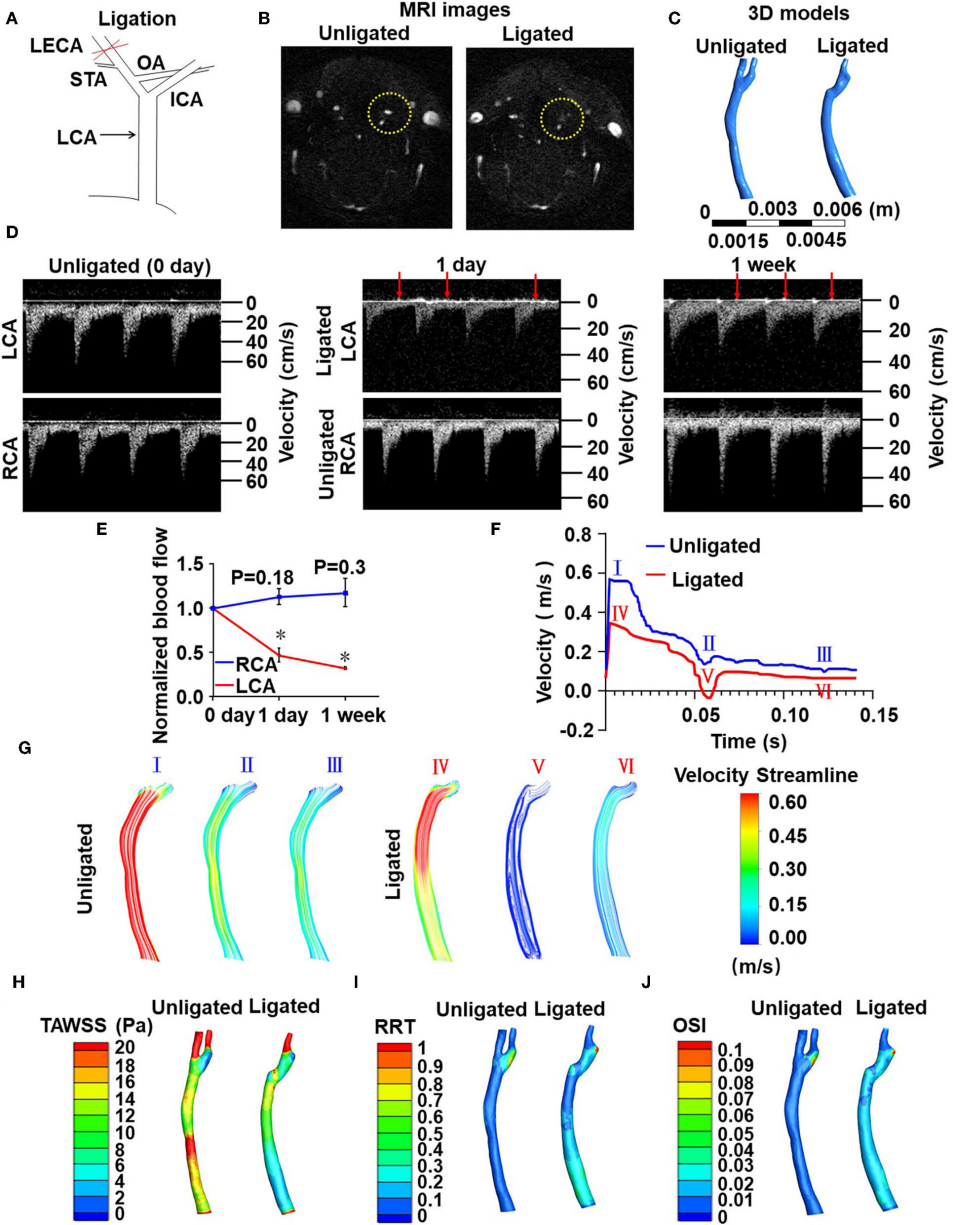
Ligation of LECA causes blood flow disturbance in the common carotid artery. **(A)** Ligation of LECA. LCA, left common carotid artery; ICA, internal carotid artery; STA, superior thyroid artery; OA, occipital artery. **(B)** Representative magnetic resonance imaging (MRI) of the neck of mice before (unligated) and after (ligated) ligation of LECA. Yellow circles indicate the LCA from unligated and ligated mouse. **(C)** 3D reconstruction of blood vessels in the neck from MRI images. **(D)** Blood flow velocity and directions of LCA and RCA were measured by high-resolution ultrasound doppler before ligation and 1 day or 1 week after ligation. Red arrows indicate reversals of blood flow. **(E)** Ligation of LECA reduces the blood flow of LCA, without significantly increasing RCA blood flow. **(F)** The velocity over a cardiac cycle in unligated and ligated mice. Peak systole (I and IV), end systole (II and V), distole (III and VI) in the pre- and post-ligation flow curves are indicated. **(G)** Images were color streamline of velocity magnitude in three time points of the pre- and post-ligation models. **(H–J)** Pseudocolor images of time-averaged wall shear stress (TAWSS) **(H)**, Relative residence time (RRT) **(I)**, and oscillation shear index (OSI) **(J)** in the common carotid arteries before and 1 week after ligation. The results represent the ratio of blood flow velocity after ligation to that before ligation. Data are mean ± SEM, *n* = 5, *P < 0.05.

To determine whether flow disturbance accelerates arterial thrombosis, we initially utilized a well-defined disturbed-flow model, partial ligation of mouse carotid artery (Nam et al., 2009), in which the external carotid, internal carotid, and occipital arteries of LCA were ligated (Supplementary Figure 1). Flow velocity and directions in LCA and the right common carotid arteries (RCA) were determined by flowmeter and high-resolution ultrasonography. As expected, these were a reduction in flow velocity and an appearance of flow reversal (indicated by arrows) in LCA at 1 day and 1 week post-ligation (Supplementary Figure 1C). The peak flow velocity in LCA dropped from 63.06 ± 12.51 cm/s (pre-ligation) to 9.17 ± 0.08 cm/s (1 day post-ligation) (Supplementary Figure 1C). Unfortunately, the very low flow velocity (Supplementary Figure 1B) forbade this model being used in combination with the FeCl_3_-induced arterial injury to study thrombus formation. We therefore modified the ligation model as followed: Only the LECA was ligated while the other branches of the LCA were preserved (Figure 1A). To determine whether ligation of the LECA causes changes in the magnitudes and directions of the wall shear stress in LCA, we performed computational fluid dynamics (CFD) modeling. MRI of mouse neck was performed before and after ligation and three-dimensional images were reconstructed to obtain the precise *in vivo* anatomy of the LCA (Figures 1B,C). The values of flow velocity, direction, and vessel dimensions of LCA and RCA were measured by high resolution ultrasonography (Figure 1D). The results showed a 50 and 62% reduction, respectively, in flow velocity during systole in LCA at 1 and 7 days after ligation (Figures 1D,E), indicating that the flow velocity was much higher than that in the original partial ligation model (Supplementary Figure 1C). There was no flow reverse in the RCA in the cardiac cycle, while in the LCA there was a significant reverse flow at the very end of systole (Figures 1D,F). Based on the flow curve measured by ultrasonography, blood flow fields and hemodynamic parameters such as flow velocity (Figures 1F,G), time-averaged wall shear stress (TAWSS) (Figure 1H), relative residence time (RRT) (Figure 1I) and oscillatory shear index (OSI) (Figure 1J) in the common carotid arteries were calculated by the CFD analysis (Figures 1F–J). In the reverse flow phase of the ligated LCA, oscillatory recirculation zones and lower flow velocity were observed in comparison with that in the unligated vessels (Figures 1F,G). Since the low and reverse flow, the lower TAWSS, higher RRT and higher OSI that have been previously suggested as athero-prone parameters appeared in the LCA (Lee et al., 2009), these results indicated that ligation of LECA causes blood flow disturbance in the common carotid artery.

### Ligation of LECA Induces Endothelial Inflammation

**Figure 2:**
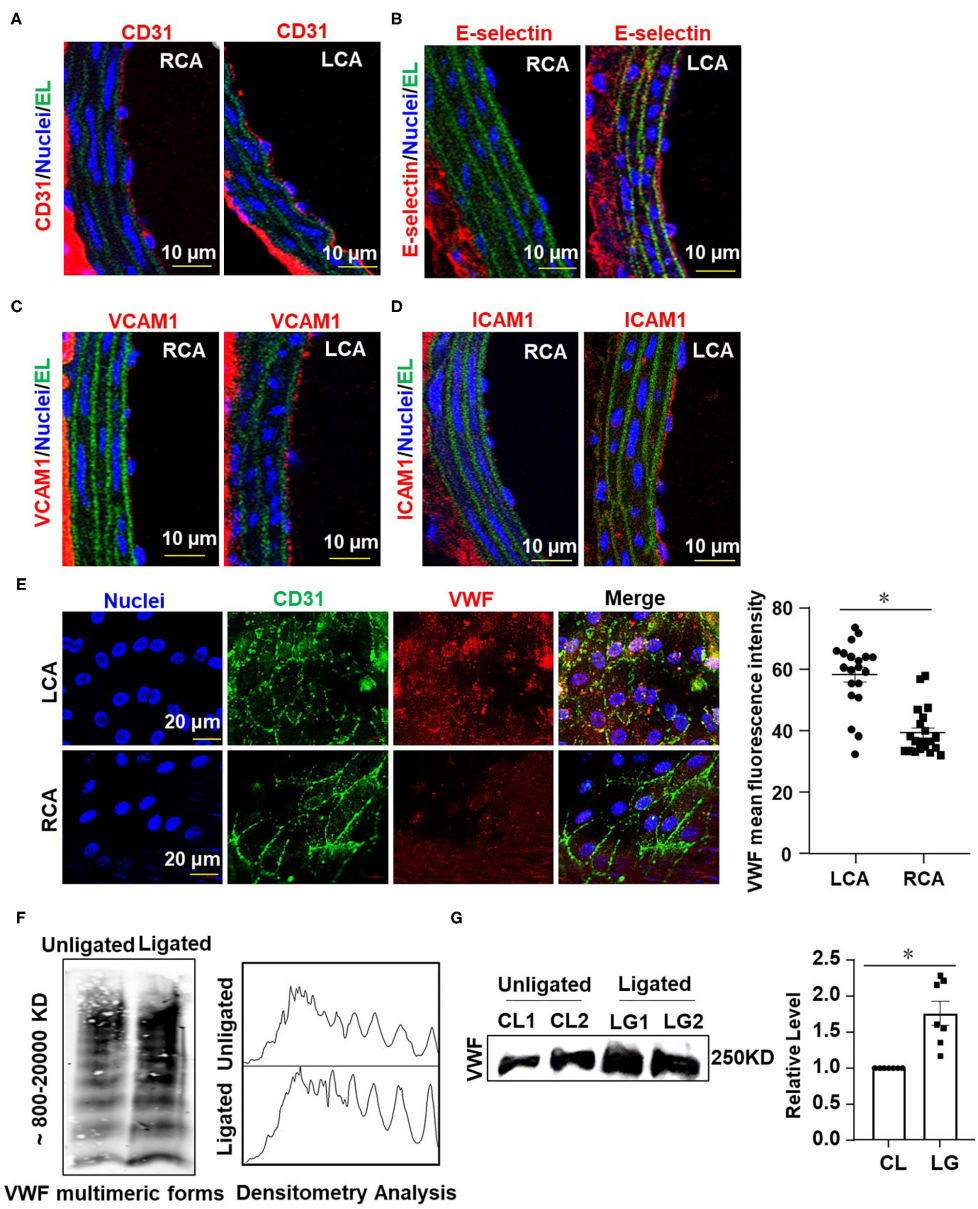
Ligation of LECA induces endothelial inflammation and VWF secretion. **(A–D)** Representative images for immunofluorescent staining of E-selectin (red), VCAM-1 (red), ICAM-1 (red), and endothelial marker CD31 (red) in the unligated RCA or the ligated LCA 1 week-post ligation. EL, elastic lamina (green), the number of tissue samples in **(A–D)** was 7 (*n* = 7). **(E)** VWF levels on the endothelial surfaces of the ligated LCA and the unligated RCA were assessed by *en face* immunofluorescence 1 day after external carotid artery ligation, and VWF mean fluorescence intensity in LCA and RCA were quantified. *n* = 20 (ligated LCA), *n* = 21 (unligated RCA). **(F,G)** The plasma level of VWF was determined at 1 day after ligation. VWF multimeric patterns and the total VWF level in plasma from the unligated or ligated mice were detected by Western blot assay followed with SDS-agarose gel electrophoresis **(F)** or SDS-polyacrylamide gel electrophoresis **(G)**. CL represented plasma was obtained from unligated mouse; LG represented plasma was derived from the ligated mice. Densitometric analysis of VWF multimers was performed (**F**, right panel) and the average gray values of VWF were analyzed quantitatively (**G**, right panel). Data are mean ± SEM, *n* = 7, *P < 0.05.

To further validate our modified disturbed-flow model, we tested whether ligation of the LECA results in functional consequences in the common carotid artery. Disturbed flow has been indicated by numerous studies to promote the expressions of pro-inflammatory adhesion molecules in vascular endothelium (Chiu and Chien, 2011). We performed immunofluorescence staining on sections of RCA and LCA from mice after 1 week of ligation to detect the expression of endothelial marker gene, cluster of differentiation 31 (CD31), and the pro-inflammatory genes E-selectin, vascular cell adhesion molecule 1 (VCAM-1) and intercellular cell adhesion molecule 1 (ICAM-1). Ligation of LECA did not lead to endothelial denudation (Figure 2A), however, it caused increases in expressions of E-selectin, VCAM-l, and ICAM-1 in the endothelia of the common carotid arteries, in comparison with the expression from the unligated RCA (Figures 2B–D). These findings indicated that ligation of the LECA does result in endothelial dysfunction, which is very likely attributable to the effects of disturbed blood flow.

### Ligation of LECA Induces the Endothelial Secretion of VWF

The hemostatic protein VWF plays a crucial role in thrombus formation. We measured the VWF levels on the intimal surface of LCA and RCA from mice at 1 day after ligation by immunofluorescent staining. The images showed that the VWF-positive staining on the surface of vascular endothelium in LCA was much greater than that in RCA (Figure 2E), implying that the secretion of VWF from endothelia is enhanced by disturbed flow. Studies have shown that traumatic vascular injury results in ultra-large VWF formation and increased plasma VWF levels (Dyer et al., 2020; Plautz et al., 2020). Therefore, we detected the levels of VWF in circulating blood plasma from mice with ligation of the LECA or those with a sham surgery by Western blot assay followed with SDS-agarose gel electrophoresis or SDS-polyacrylamide gel electrophoresis (Figure 2F, left panel and Figure 2G). In plasma, VWF appeared as a series of multimers of regularly decreasing molecular mass from several thousand to hundreds kDa that could be separated and distinguished by SDS-agarose gel electrophoresis but not SDS-polyacrylamide gel electrophoresis. Densitometric analysis of VWF multimers was performed with Image J software according to VWF multimers electrophoretic gel images. Optical density values representing the concentrations of VWF multimers of different molecular weights were processed as grayscale data (Studt et al., 2001). The analysis confirmed the presence of high- and middle-molecular-weight VWF multimers (Figure 2F, right panel). The largest VWF multimers contain multiple sites for interaction with platelets and vessel wall components and are therefore thought to have a greater thrombogenic potential (Ruggeri, 2001). The results from the two gel electrophoreses indicated that ligation of LECA promotes VWF secretion.

### Ligation of LECA Accelerates Thrombosis After the FeCl_3_-Induced Arterial Injury

**Figure 3:**
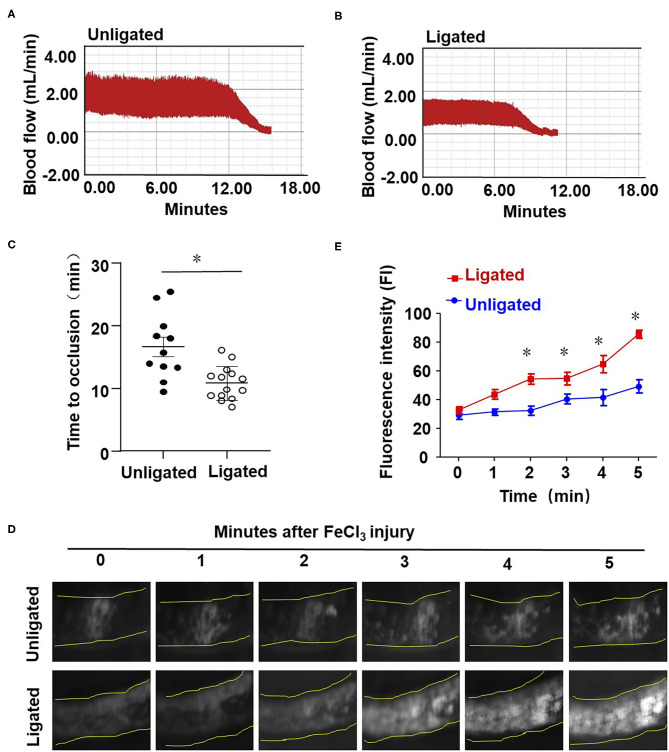
Ligation of LECA accelerates thrombosis upon FeCl_3_-induced arterial injury. **(A,B)** The LECA of C57BL/6 mice were ligated or unligated (sham operation), and blood flow in the LCA following 20% FeCl_3_ induced injury was monitored by a doppler echocardiogram. The FeCl_3_ injury-induced thrombosis assay was performed at 1 day after ligation. Representative image is the timeline starting immediately from placement of the 20% FeCl_3_ filter paper to the time to occlusion of the unligated **(A)** or ligated mice **(B)**. **(C)** The time of thrombus formation in unligated and ligated mice was analyzed quantitatively, *n* = 11 (unligated mice), *n* = 14 (ligated mice). **(D)** Rhodamine 6G was injected into the right jugular vein of unligated and ligated mice to label platelets, and platelet adhesion to LCA was monitored immediately upon application of 20% FeCl_3_ (1 day after ligation). Images showed platelet adhesion and thrombus formation in carotid arteries within the indicated durations (3 min) after the application of 20% FeCl_3_. **(E)** Comparison of the fluorescence intensity in FeCl_3_-treated arteries from unligated and ligated mice, *n* = 5 (unligated mice), *n* = 5 (ligated mice). Data are mean ± SEM, *P < 0.05.

To study the influence of disturbed flow on arterial thrombus formation *in vivo*, we combined the ligation of LECA with FeCl_3_-induced arterial injury, the latter is a well-established technique to induce the formation of thrombi rapidly and accurately in an exposed artery (Kurz et al., 1990; Farrehi et al., 1998). FeCl_3_-induced thrombus formation was performed in mouse RCA and the ligated LCA or carotid arteries which had been previously subjected to ligation (ligated) or sham surgery (unligated). When the comparison was conducted between the RCA and LCA in one mouse, the average time to occlusion in RCA was 28.75 ± 7.63 min, while in LCA it was reduced to 11.23 ± 2.73 min which was significantly shorter than that in the unligated vessels (Supplementary Figure 2). When the sham or ligated operated mice were compared, the average times to occlusion were 16.67 ± 5.16 and 10.87 ± 2.69 min, respectively (Figures 3A–C). To further validate the role of flow disturbance in pro-thrombosis, real-time observation of carotid artery thrombi formation was performed. In this assay, rhodamine 6G solution was injected into the right jugular vein to label the circulating platelets, as previously described (Li et al., 2016). *In vivo* fluorescence microscopy of the common carotid artery showed that the platelets labeled with rhodamine 6G accumulated along the vessel wall immediately upon 20% FeCl_3_ treatment, and thrombi formation was observed in all mice 1 min upon injury (Figure 3D). The initially formed thrombi were unstable and parts of them were usually washed away by the blood flow, so the formed thrombi did not enlarge in the unligated vessels (Figures 3D,E). By contrast, there was a significantly accelerated thrombi formation and stabilization duration in the ligated vessels, in which thrombi started to enlarge from 3 to 4 min after removing the filter paper with FeCl_3_ and usually filled the vessel segment within 5 min (Figures 3D,E). These results provide *in vivo* evidence demonstrating that disturbed flow promotes thrombosis.

### Oscillatory Shear Enhances Endothelial VWF Secretion and Provokes Platelet Aggregation

**Figure 4:**
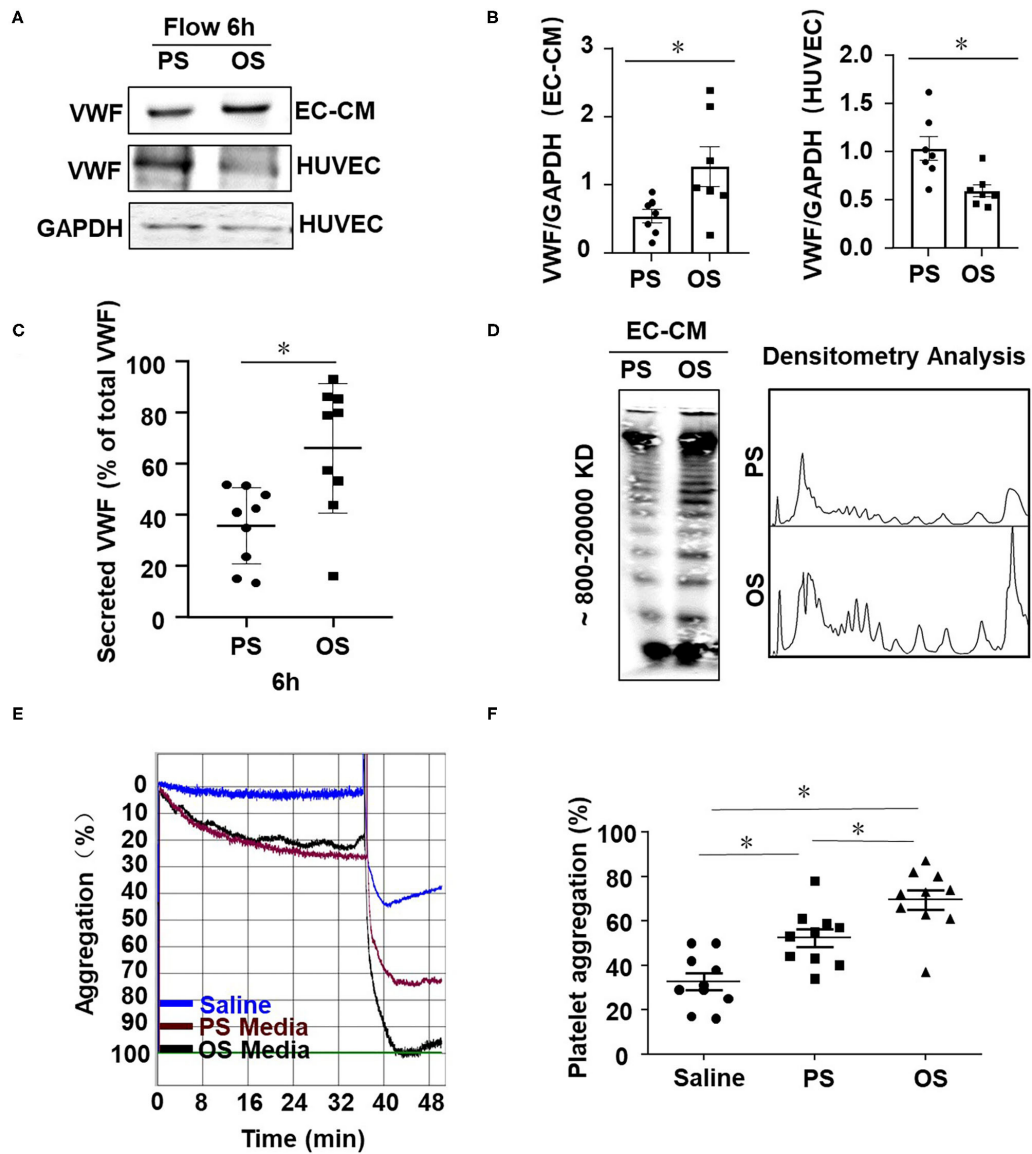
Oscillatory shear enhances endothelial VWF secretion and provokes platelet aggregation. **(A)** Cells were exposed to PS (12 ± 4 dynes/cm^2^) or OS (0.5 ± 4 dynes/cm^2^) for 6 h and the expressions of VWF in the conditioned media and cells were analyzed by Western blot assay followed with SDS-polyacrylamide gel electrophoresis. **(B)** The average gray values of VWF in **(A)** were analyzed quantitatively. **(C)** The secreted VWF in conditioned media were detected by ELISA, *n* = 9 (PS), *n* = 9 (OS). **(D)** VWF multimers were analyzed by Western blot assay followed with SDS-agarose gel electrophoresis and densitometric analysis. **(E)** Representative curves of human platelet aggregation assay. The platelet-rich plasma (PRP) were mixed with saline solution or the conditioned media from the sheared endothelial cells and platelet aggregation was induced by adding ADP into the mixture. **(F)** Platelet aggregation is expressed as the percentage of light transmittance (y-axis) over time (x-axis). Platelet aggregation in each group were recorded and quantitatively analyzed using one-way ANOVA. *n* = 10 (Saline), *n* = 10 (PS), *n* = 10 (OS). Data are mean ± SEM, *P < 0.05.

Next, we examined the influences of shear stress with distinct flow patterns on endothelial cell VWF secretion and platelet aggregation at the cellular level. OS at 0.5 ± 4 dynes/cm^2^ was used to mimic the fluid shear stress at regions downstream of the stenotic wall and near the reattachment point, where low (near-zero) and oscillatory wall shear stress occurs (Hyun et al., 2000). PS at 12 ± 4 dynes/cm^2^ derives from a typical laminar flow at straight part of the main arteries (Hathcock, 2006). Application of OS to endothelial cells for 6 h markedly increased the level of VWF in the conditioned media, as compared with the application of PS (Figures 4A,B, left and Figure 4C). The conditioned media were collected from the endothelial cell culture (an incubation for 30 min) with pre-exposure to PS or OS for 6 h. OS appeared to reduce the intracellular VWF levels compared with PS (Figures 4A,B, right panel), which might be due to the increase in VWF secretion. VWF multimers in the conditioned media were analyzed by SDS-agarose gel electrophoresis, followed by densitometric analysis. The results indicated that OS promoted the secretion of high-molecular-weight VWF multimers, as compared with PS (Figure 4D). To examine the role of fluid shear stress applied to endothelial cells in hemostasis, we evaluated the influences of endothelial cell conditioned media on platelet aggregation induced by ADP in PRP. Conditioned media from the indicated treatments were mixed with PRP from human whole blood and platelet aggregator was used to detect platelet aggregation. The aggregation rate in conditioned media from cells with OS was significantly higher than that with PS (Figures 4E,F). These results suggest that oscillatory shear stress enhances endothelial VWF secretion and provokes platelet aggregation.

### Oscillatory Shear Stress Enhances Endothelial VWF Secretion via Promoting Activation of VAMP3 and SNAP23

**Figure 5:**
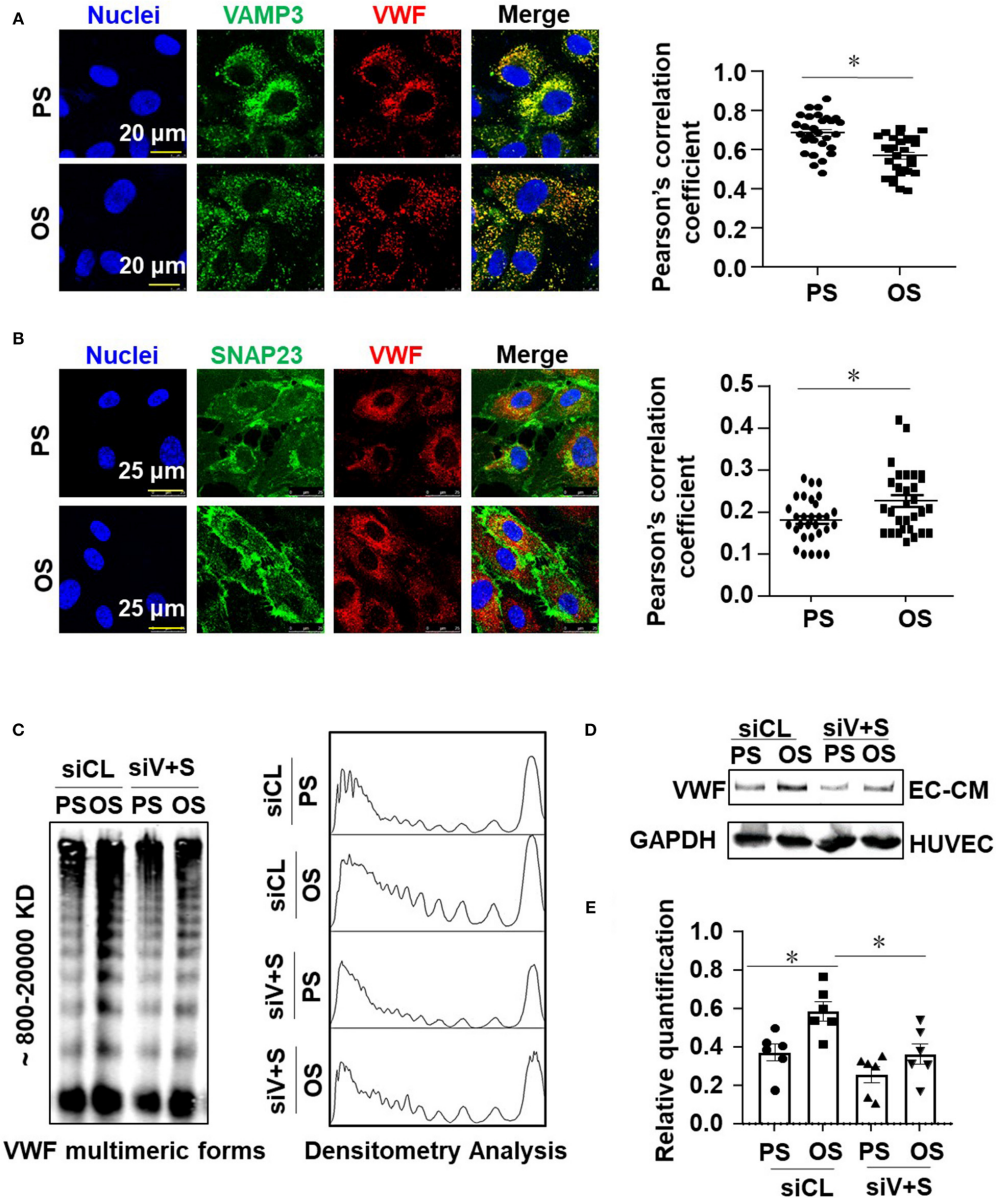
Oscillatory shear stress enhances endothelial VWF secretion via promoting the activation of VAMP3 and SNAP23. **(A,B)** Cells were exposed to PS (12 ± 4 dynes/cm^2^) or OS (0.5 ± 4 dynes/cm^2^) for 1 h, and then the subcellular localizations of VAMP3, SNAP23, and VWF were assessed. The colocalization of VAMP3 and VWF **(A)**, SNAP23 and VWF **(B)** were quantified. **(C,D)** Cells were transfected with siRNAs targeting VAMP3 (siV) and SNAP23 (siS) or the control siRNAs at a concentration of 40 nmol/L, and were then exposed to PS or OS for 6 h. **(C)** The VWF multimers, and **(D)** the total levels of VWF in conditioned media were assessed. **(E)** The bands from SDS-polyacrylamide gel electrophoresis were quantified. Results in **(E)** are mean ± SEM from **(D)**, *n* = 6, *P < 0.05.

Our previous research has demonstrated the disturbed flow- or oscillatory shear stress-activated VAMP3 and SNAP23 expressions and association in vascular endothelial cells (Zhu et al., 2017). Here we sought to investigate the role of VAMP3 and SNAP23 in OS-evoked VWF secretion. Cells were subjected to PS or OS for 1 h and the subcellular localizations of VAMP3, SNAP23, and VWF were detected. In contrast to previous study in which prolonged shear stress load (6 or 24 h) was utilized, in the current mechanistic study we were focusing on short-term regulation to avoid the effects of shear stress on expressions of VAMP3 and SNAP23 (Supplementary Figure 3). The subcellular localizations of VAMP3 and SNAP23 were distinct in cells subjected to PS or OS. The most predominant phenotype for VAMP3 in cells under PS was perinuclear localization with a condensed appearance, whereas in cells under OS it exhibited a dispersed appearance (Supplementary Figure 4). SNAP23 was located mainly in the plasma membrane and also in scattered cytoplasmic structures in cells exposed to PS; in comparison, it was intensively localized to the plasma membrane following OS exposure (Supplementary Figure 4). This phenomenon is in line with our previous findings (Zhu et al., 2017). Quantification of the degree of colocalization between fluorophores using Pearson's correlation coefficient indicated an increased co-localization between VAMP3 and SNAP23 in OS vs. PS (Supplementary Figure 4), suggesting an enhancement of SNARE-mediated WPB fusion with the plasma membrane. The colocalization of VAMP3 with VWF was actually a bit lower in OS than PS (Figure 5A). Since VAMP3 is mainly located at WPB membrane, the colocalization of VAMP3 with VWF might decrease once the release of VWF is activated by OS. The colocalization of SNAP23 with VWF was found higher in OS than PS (Figure 5B). The results suggest that more WPBs fuse with cell membrane in OS.

To determine whether VAMP3 and SNAP23 mediate the OS-induced VWF secretion, cells transfected with siRNAs targeting VAMP3 or/and SNAP23 or the control siRNA (Supplementary Figure 5) were subjected to PS or OS for 6 h and VWF secretion was then examined. Western blot assay demonstrated a compromised constitutive or OS-induced secretion of VWF in the VAMP3 and SNAP23 inhibited cells (Figures 5C–E and Supplementary Figure 5). Taken together, these results validated the role of VAMP3 and SNAP23 in mediating the OS-evoked VWF secretion.

### Vimentin Associates With SNAP23 to Modulate the Oscillatory Shear Stress-Induced Translocation of SNAP23 to the Cell Membrane

**Figure 6:**
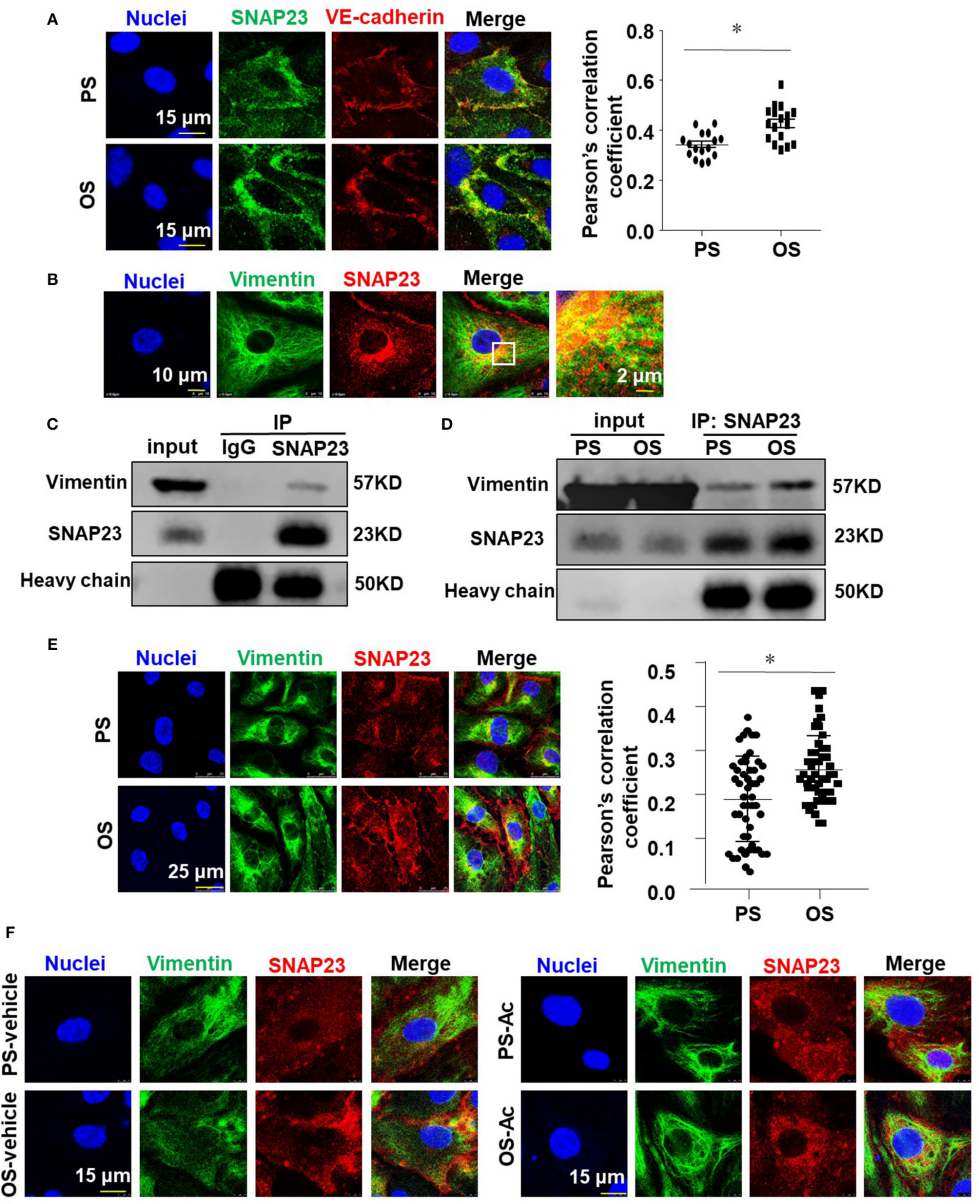
Vimentin associates with SNAP23 to modulate the oscillatory shear stress-induced translocation of SNAP23 to the cell membrane. **(A,B,E)** Cells were exposed to PS (12 ± 4 dynes/cm^2^) or OS (0.5 ± 4 dynes/cm^2^) for 1 h **(A,E)** or kept in static condition **(B)**, and then the subcellular localizations of SNAP23, VE-cadherin and vimentin were assessed by immunofluorescence. Pearson's correlation coefficient was calculated to quantify their colocalizations. **(C,D)** Cells were exposed to PS or OS for 1 h **(D)** or kept in static condition **(C)**, and the association of SNAP23 with vimentin was assessed by co-IP (IP: SNAP23, IB: Vimentin). **(F)** Cells were pretreated with acrylamide (Ac, 4 mM) or with the control vehicle (H_2_O) for 16 h, exposed to PS or OS for 1 h, and the subcellular localizations of vimentin and SNAP23 were assessed. Numbers of quantified cells or fields in each experiment are as followed: in **(A)**, *n* = 17 (PS), *n* = 17 (OS); in **(E)**, *n* = 50 (PS), *n* = 50 (OS). Results in **(A,E)** are mean ± SEM, *P < 0.05.

To explore that how OS activates SNAP23, cells were exposed to PS or OS for 1 h and the translocation of SNAP23 to the cell membrane induced by OS was confirmed by immunofluorescence-based colocalization analysis of SNAP23 and vascular endothelial cadherin (VE-cadherin), which is localized at adherent junctions (Figure 6A). It has been reported that SNAP23 is targeted to vimentin filaments in fibroblasts and that transfer of SNAP23 from the vimentin-associated reservoir to functional plasma membrane pool may modulate the availability of SNAP23 to form SNARE complexes (Faigle et al., 2000). This prompted us to hypothesize that vimentin associates with SNAP23 in endothelial cells to modulate the OS-induced translocation of SNAP23 to the plasma membrane. To test this hypothesis, we first verified the colocalization of vimentin with SNAP23 in endothelial cells without shearing by immunofluorescent staining, which suggests a potential constitutive association between vimentin and SNAP23 (Figure 6B). To validate a physical interaction between vimentin and SNAP23, co-IP assay was performed (Figures 6C,D). The results revealed that compared with the control IgG, the SNAP23 antibody-precipitated immunocomplexes were vimentin enriched, suggesting a constitutive physical interaction between vimentin and SNAP23 (Figure 6C). Their association could be enhanced by OS exposure vs. PS (Figure 6D). The OS-promoted interaction between vimentin and SNAP23 was further confirmed by immunofluorescent staining (Figure 6E and Supplementary Figure 6) and proximity ligation assay determination of these two proteins (Supplementary Figure 7). It should be noted that shear stress altered the cellular localization of vimentin, as vimentin filaments redistributed to the adjacent areas of the cortex during exposure to OS (Figure 6E). To disrupt vimentin, cells were treated with monomeric acrylamide at a final concentration of 4 mM (Haudenschild et al., 2011) or control reagent and were then subjected to PS or OS. Treatment with acrylamide resulted in a condensed and perinuclear distribution of vimentin, indicating that the normal organization and function of vimentin networks may have been impaired (Supplementary Figure 8A). In the control treatments, OS promoted the plasma membrane-localization of vimentin and SNAP23, in comparison with PS (Figure 6F, left panels). Treatment with acrylamide not only prevented the redistribution of vimentin but also prohibited the translocation of SNAP23 to the cell membrane in cells with OS (Figure 6F, right panels and Supplementary Figure 8B). Altogether, these findings suggest that OS induces movement of vimentin intermediate filaments to mediate the translocation of SNAP23.

### Targeting VAMP3 and SNAP23 Could Influence the Disturbed Flow-Induced Thrombosis

**Figure 7:**
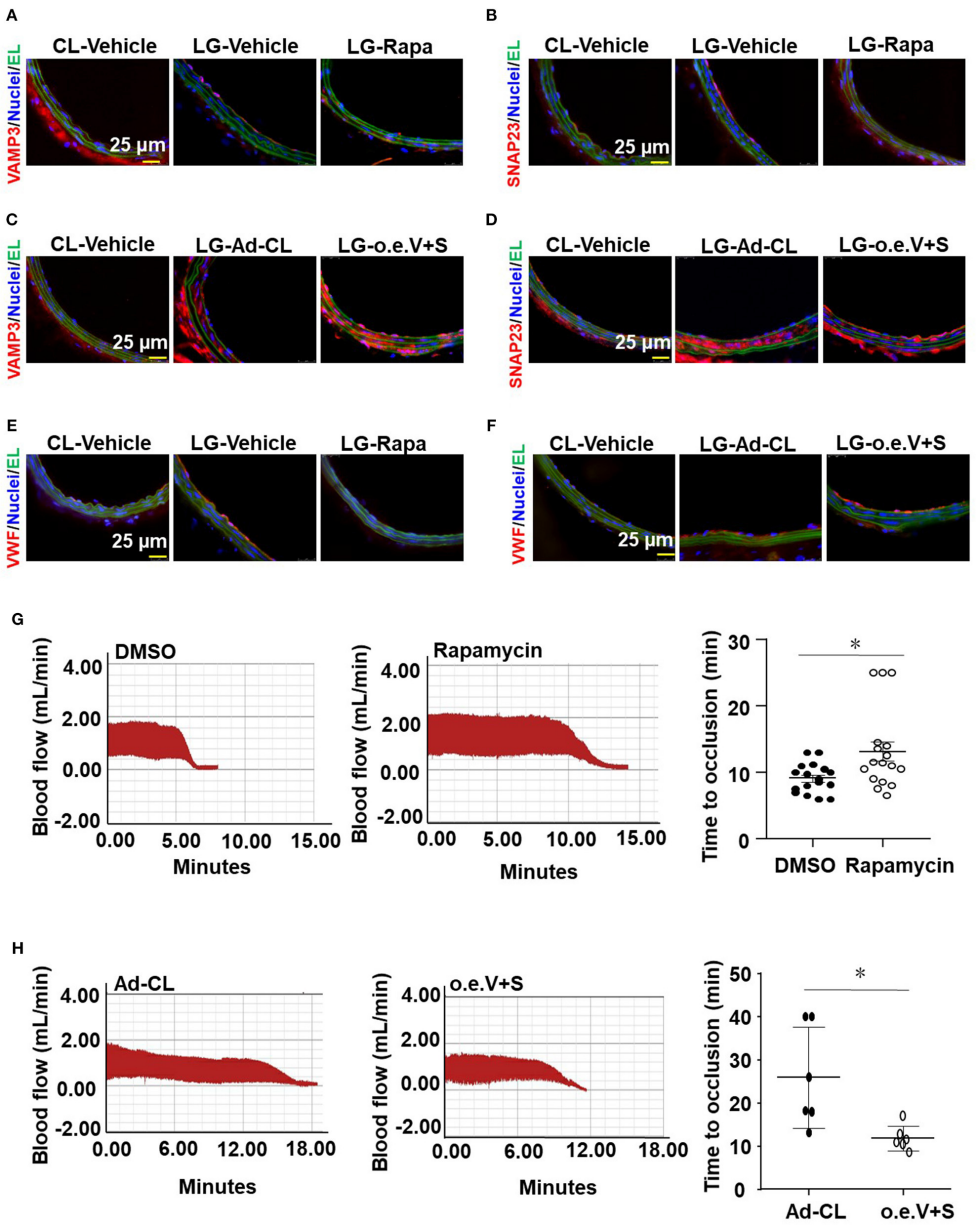
VAMP3 and SNAP23 play important roles in disturbed flow-induced thrombosis. C57BL/6 mice were subjected to ligation of LECA. Some mice were then subjected to intraperitoneal injection with rapamycin (Rapa, 1 mg/kg body weight) or the control vehicle (DMSO) every other day for 7 days **(A,B)**, others were subjected to a locally intraluminal incubation with adenovirus expressing VAMP3(Ad-VAMP3) and SNAP23(Ad-SNAP23) or the control virus (Ad-CL) **(C,D)**. **(A–F)** Representative images of immunofluorescent staining of VAMP3, SNAP23 and VWF in unligated RCA or ligated LCA of mice. CL, the unligated RCA; LG, the ligated LCA; EL, Elastic lamina; o.e.V+S, Ad-VAMP3 and Ad-SNAP23. **(G,H)** LCA of the ligated mice were then subjected to FeCl_3_-induced arterial injury. Blood flows in the LCA were monitored by a doppler echocardiogram. Representative images are the timeline starting immediately from placement of the FeCl_3_ filter paper to time to occlusion of the carotid artery occlusion. **(G,H)** Quantification and statistical analysis of the time to occlusion. Data are mean ± SEM, *n* = 17 per group **(G)**, *n* = 6 per group **(H)**, * *P* < 0.05.

To clarify the role of VAMP3 and SNAP23 in mediating the disturbed flow-accelerated thrombosis *in vivo*, we performed loss- and gain-of-function studies of VAMP3/SNAP23 in mice with ligation of the LECA followed by FeCl_3_-induced arterial injury. Our previous studies have shown that systemic delivery of mTOR complex 1 (mTORC1) inhibitor, rapamycin, can reduce the expressions of VAMP3 and SNAP23 in arterial endothelium (Zhu et al., 2017). Thus, to inhibit VAMP3 and SNAP23, mice were either received intraperitoneal injection of rapamycin or its control solvent in current experiments. For the gain-of-function of VAMP3/SNAP23, the LCAs of mice were subjected to a local intraluminal incubation of adenovirus expressing SNAP23/VAMP3 (Ad-SNAP23 and Ad-VAMP3) or its control virus before ligation. As expected, ligation of LECA resulted in increased expressions of VAMP3 and SNAP23 in the endothelial of the common carotid arteries at 1 week post-surgery. The increase could be suppressed by systemic delivery of rapamycin or be further enhanced by intraluminal application of Ad-VAMP3 and Ad-SNAP23 viruses (Figures 7A–D). Treatment with rapamycin significantly reduced VWF level on the intimal surface. In contrast, intraluminal application of Ad-SNAP23 and Ad-VAMP3 viruses significantly increased VWF level on the intimal surface (Figures 7E,F). Treatment with rapamycin significantly ameliorated the FeCl_3_-induced thrombogenesis in the carotid arteries of mice with average time to occlusion extended from 9.12 ± 2.2 min to 13.17 ± 6.07 min (Figure 7G). On the contrary, intraluminal application of Ad-SNAP23 and Ad-VAMP3 viruses significantly exaggerated FeCl_3_-induced thrombogenesis in the treated arteries in compared with those in the controls (Figure 7H). The average time to occlusion was reduced from 25.88 ± 11.68 min to 11.84 ± 2.88 min (Figure 7H). Our results suggest VAMP3 and SNAP23 as potential targets for preventing the disturbed flow-accelerated thrombus formation.

## Discussion

Atherosclerotic geometries-resulted fluid mechanical conditions have been suggested to promote platelet aggregation and thrombus formation (Nesbitt et al., 2009). To study the phenomenon and the underlying mechanisms, sophisticated and dedicated research models have been developed and applied. In Nesbitt et al. (2009) study, the vessel geometry as well as local blood flow were altered by mechanically compressing the vessel with a blunted microinjection needle to progressively stenose the vessel lumen. CFD modeling of the stenotic vessel geometry revealed generation of disturbed blood flow downstream of the stenotic site. Similarly in Westein et al. (2013) study, stenosis was induced by localized vessel compression with a 27-gauge needle. Synergetic effects of atherosclerotic geometries and vessel injury on platelet aggregation and thrombosis were investigated on the basis of the disturbed flow model mentioned above in combination with photoactivation with an excitation wavelength at 550 nm or microinjection of 6% FeCl_3_ into the tissue adjacent to the arteriole of interest (Nesbitt et al., 2009). Although these studies indicate that flow disturbance is associated with thrombus formation, they do not provide evidence showing how disturbed flow affects the endothelium-dependent regulation on thrombosis and hemostasis. Another limitation of the mechanical compression model is that the areas exposed to disturbed flow are very small which makes it difficult to analyze endothelial gene expression and function in those areas.

Partial ligation of carotid artery was initially described as a model of flow reduction to study vascular remodeling (Sullivan and Hoying, 2002). In this model, all branches originating from LCA, except for the left thyroid artery, were ligated, resulting in a substantial flow reduction in LCA. By using CFD modeling incorporated the geometry of mouse arteries as determined by high-resolution ultrasound measurements, Nam et al. described partial carotid ligation as a model of disturbed flow with characteristics of low and oscillatory wall shear stress (Nam et al., 2009). In response to partial ligation, blood flow significantly decreased (−90%) in LCA. The substantial local application of FeCl_3_ might induce thrombus formation in the operated LCA as well as decrease and stagnation of blood flow as the flowmeter would detected. Since the assessment of thrombosis is based on the time to cessation of blood flow, it becomes very difficult to determine a further decrease if the flow rate is compared with an extreme low baseline. We therefore modified the partial ligation model. In our adapted model only the LECA is ligated, resulting in a generation of low and oscillatory shear stress in the LCA. CFD simulation on the basis of vessel geometry and flow velocity provided by MRI and high-resolution ultrasound measurements revealed decreases in blood flow velocity and TAWSS, and increases in OSI and RRT in LCA in comparison with pre-ligation (Figures 1E–J), suggesting a successful generation of disturbed flow. To note that the flow velocity in the ligated LCA is appropriate 20–30 cm/s, which is much higher than that in the partially ligated LCA in previous model. To validate the functional consequences of disturbed flow applying to the vessel wall, expressions of pro-inflammatory factor E-selectin, VCAM-1 and ICAM-1 in the common carotid artery endothelial cells were measured and verified an induction of endothelial inflammation (Figures 2B–D). These results agree with previous reports showing the partial ligation-induced endothelial dysfunction (Nam et al., 2009).

Regulations of fluid shear stress on expression and secretion of VWF have been studied previously by the utilization of various *in vitro* models. Using a parallel plate flow apparatus, Hough et al. found that exposure of endothelial cells to laminar shear stress at 15 dynes/cm^2^ for 6 or 24 h enhanced VWF promoter activity to increase the mRNA level of VWF (Hough et al., 2008). By the utilization of the same flow apparatus, Gomes et al. reported that exposure of endothelial cells to laminar shear stress at 13 dynes/cm^2^ for 2 h promoted the secretion of VWF (Gomes et al., 2005) with no mechanistic explanations were mentioned. Similar results reported by Galbusera et al. using a cone-and-plate flow device showed that exposure of endothelial cells to laminar shear stress of varying magnitude (from 2 to 12 dynes/cm^2^) for 6 h increased the VWF secretion in a magnitude dependent manner (Galbusera et al., 1997). It should be noted that in the above studies the controls were set to be static condition, which may not be physiologically relevant in the study of shear-modulated endothelial function. To the best of our knowledge, this present work is the first study to compare the regulation of OS vs. PS on VWF secretion.

Vesicular-transport protein VAMP3 was originally found to be enriched in the membrane of GluT4 secretory granules and undergoes translocation form the cytoplasmic subcompartment to the plasma membrane in response to insulin to mediate glucose transport in adipocyte (Cain et al., 1992). In endothelial cells, VAMP3 was found localized on the surface of WPBs, cooperated with SNAP23 that are localized predominantly to the plasma membrane to direct the fusion of WPBs with the plasm membrane, and thus mediating the exocytosis of VWF (Pulido et al., 2011). Enhancement in the on-WPBs-surface-localization of VAMP3 and on-plasma-membrane-localization of SNAP23 would increase the chances of an encounter with each other, allowing aggravated WPB exocytosis and the consequently promoted VWF secretion happen. In previous study we have shown that disturbed flow increased the endothelial secretion of athero-prone microRNAs via the activation of VAMP3 and SNAP23 at both transcriptional and translocational levels; pharmacological intervention of mTORC1 inhibited the transcription of both VAMP3 and SNAP23. In the current study we extended our research and explored the mechanisms by which disturbed flow or oscillatory shear stress damage the endothelium to initiate platelet capture and thrombus formation. Our study has fully proved that flow disturbance accelerates the FeCl_3_-induced thrombosis and the effects are dependent on the VAMP3 and SNAP23-mediated VWF secretion (Figures 3–5, 7). Our study showed that knockdown of VAMP3 and SNAP23 could compromise the flow disturbance-evoked endothelial VWF secretion (Figures 5C–E), providing direct evidence demonstrating the contribution of endothelial VAMP3/SNAP23 to thrombosis. Notably the *in vivo* intervention of VAMP3/SNAP23 by systemic delivery of rapamycin ameliorates thrombi formation (Figure 7G), echoing the value of mTOR inhibition in preventing the development of atherosclerosis (Kurdi et al., 2016; Zhu et al., 2017). Furthermore, we provided mechanistic evidence showing that how disturbed flow activates the translocation of SNAP23 (Figure 6). Vimentin is the major and important intermediate filament protein that makes up cytoskeletons (Dave and Bayless, 2014). It is widely expressed in endothelial cells, mesenchymal cells, leukocytes, neurons cell and some epithelial cell to regulate cell adhesion, migration, secretion, and other fundamental cellular behaviors (Ikawa et al., 2014; Antfolk et al., 2017; Costigliola et al., 2017). Rapid displacement and re-organization of vimentin intermediate filaments have been observed in living endothelial cells exposed to laminar shear stress at 12 dynes/cm^2^ (Helmke et al., 2000). However, how the organization/distribution of vimentin networks response to OS vs. PS had not been characterized before. Our results revealed that OS promoted a distribution of vimentin near the cell cortex (Figures 6E,F). The subcellular localization of vimentin under OS that we observed is consistent with previous study showing that vimentin presents in endothelial cell apical cortex to provide mechanical supply to the cell (Pesen and Hoh, 2005). Vimentin filaments also interact with actin at the cell cortex to coordinate cell growth and division (Duarte et al., 2019). They may also coordinate the endothelial secretion of VWF, as our data have shown that vimentin physically interacts with SNAP23 to mediate the OS-induced translocation of SNAP23 to the cell membrane (Figure 6 and Supplementary Figure 7), and probably the consequent SNAP23-dependent fusion of WPBs with the plasma membranes. Our findings together with reports by others lead to a conclusion that vimentin is essentially involved in the mechanical force-elicited stress responses. Future studies are warranted to determine the mechanisms by which disturbed flow activatesVAMP3.

## Data Availability Statement

The original contributions presented in the study are included in the article/Supplementary Materials, further inquiries can be directed to the corresponding author/s.

## Ethics Statement

The animal study was reviewed and approved by Biomedical Ethics Committee of Peking University.

## Author Contributions

JZ, J-JZ, and Z-TJ designed study. J-JZ and Z-TJ performed research. J-JZ, Z-TJ, CL, Y-FX, JW, F-FY, W-JY, WP, L-LH, and Y-HZ discussed the data. J-JZ, A-QS, JZ, and Z-TJ analyzed data. JZ, A-QS, J-JZ wrote the paper. All authors contributed to the article and approved the submitted version.

## Conflict of Interest

The authors declare that the research was conducted in the absence of any commercial or financial relationships that could be construed as a potential conflict of interest.
