# Supplementary material for: VAMP3 and SNAP23 as Potential Targets for Preventing the Disturbed Flow-Accelerated Thrombus Formation
Source: Front Cell Dev Biol. 2020 Nov 5;8:576826. doi: 10.3389/fcell.2020.576826 (PMC7674309; doi:10.3389/fcell.2020.576826)
Supplement: Supplementary file 1 [file Data_Sheet_1.pdf]

Supplementary Material

Supplementary Figure 1. Measurements of blood flow in common carotid arteries in the partial ligation model. (A) Schematic diagram of the partial carotid ligation model, in which the left external carotid artery (LECA), internal carotid artery (ICA) and occipital artery (OA) are ligated while the thyroid artery (TA) is retained. (B) Blood flow in the left common carotid (LCA) in the partial ligation model was detected by flowmeter. The mean blood flow of the LCA was about 0.2 mL/min. (C) The velocity and direction of blood flow in the LCA and RCA were measured by high-resolution ultrasound doppler before ligation and one day or one week after ligation. The blood flow velocity of LCA was close to 0 cm/s at 1 day and 1 week after ligation. Red arrows indicate appearance of blood flow reversal.

Supplementary Figure 2. Ligation of LECA significantly reduced the average time to occlusion. (A, B) Representative images of thrombus formation. Thrombosis was induced by application of $FeCl_3$ to the mouse LCA, which has been previously subjected to ligation of the external carotid artery. Blood flow in the LCA or RCA in one mouse was monitored by a doppler echocardiogram. Blood flow droping down to about 5% of the original flow rate is considered occluded. The time to occlusion was recorded. (C) Quantification of the time to occlusion in the LCA and RCA. n = 4 (Unligated-R), n = 16 mice (Ligated-L). Data are mean ± SEM, $^*P < 0.05$.

Supplementary Figure 3. Influences of shear stress on the expressions of SNAP23 and VAMP3 in endothelial cells. Endothelial cells were exposed to PS or OS for 1 hour, SNAP23 and VAMP3 expressions in the sheared endothelial cells were detected by Western blot.

Supplementary Figure 4. Oscillatory shear stress enhances the colocalization of SNAP23 and VAMP3. Endothelial cells infected with SNAP23-GFP virus exposed to PS (12 ± 4 dynes/cm^2) or OS (0.5 ± 4 dynes/cm^2) for 1 hour, and then the subcellular localizations of VAMP3 and SNAP23 were assessed. Pearson's correlation coefficient was calculated to quantify their colocalizations, Data are mean ± SEM, n = 38 (PS), n =38 (OS), * $P < 0.05$.

Supplementary Figure 5. Knockdown of VAMP3 and SNAP23 inhibits VWF secretion. Endothelial cells were transfected with siRNAs targeting VAMP3 (siV), SNAP23 (siS), VAMP3 and SNAP23 (siV+S) or the control (siCL) siRNAs at a concentration of 40 nmol/L respectively. The efficiency of siRNA-mediated knockdown on SNAP23 and VAMP3 and the level of VWF in the conditioned media was analyzed by Western blot assay followed with SDS-polyacrylamide gel electrophoresis.

Supplementary Figure 6. Effect of PS or OS on colocalization of SNAP23 and vimentin in endothelial cells. Endothelial cells were exposed to PS or OS for 1 hour and then the subcellular localizations of SNAP23 and vimentin were assessed.

Supplementary Figure 7. Oscillatory shear stress promotes the association of SNAP23 and vimentin in endothelial cells. (A) Endothelial cells were exposed to PS or OS for 1 hour, and the association between SNAP23 and vimentin was analyzed by Proximity Ligation Assay (PLA). Red

spots correspond to SNAP23–vimentin interaction couples. (B) Quantification of (A), Each symbol represents one field. Data are mean ± SEM, n = 16 (PS), n =16 (OS), * $P < 0.05$.

Supplementary Figure 8. Treatment with acrylamide disrupts the organization of vimentin intermediate filaments as well as the cell membrane localization of SNAP23 both in static and the sheared endothelial cells. (A) Endothelial cells were treated or untreated with 4 mM Acrylamide for 16 hours, then the localization of vimentin in endothelial cells was detected by immunofluorescence staining. (B) Endothelial cells were treated with 4 mM Acrylamide for 16 hours, and then exposed to PS or OS for 1 hour, the localization of SNAP23 and VE-cadherin in Endothelial cells was detected by immunofluorescence staining.
