# Supplementary material for: VAMP3 and SNAP23 as Potential Targets for Preventing the Disturbed Flow-Accelerated Thrombus Formation
Source: Front Cell Dev Biol. 2020 Nov 5;8:576826. doi: 10.3389/fcell.2020.576826 (PMC7674309; doi:10.3389/fcell.2020.576826)
Supplement: Supplementary file 2 [file Image_1.PDF]

Supplementary materials

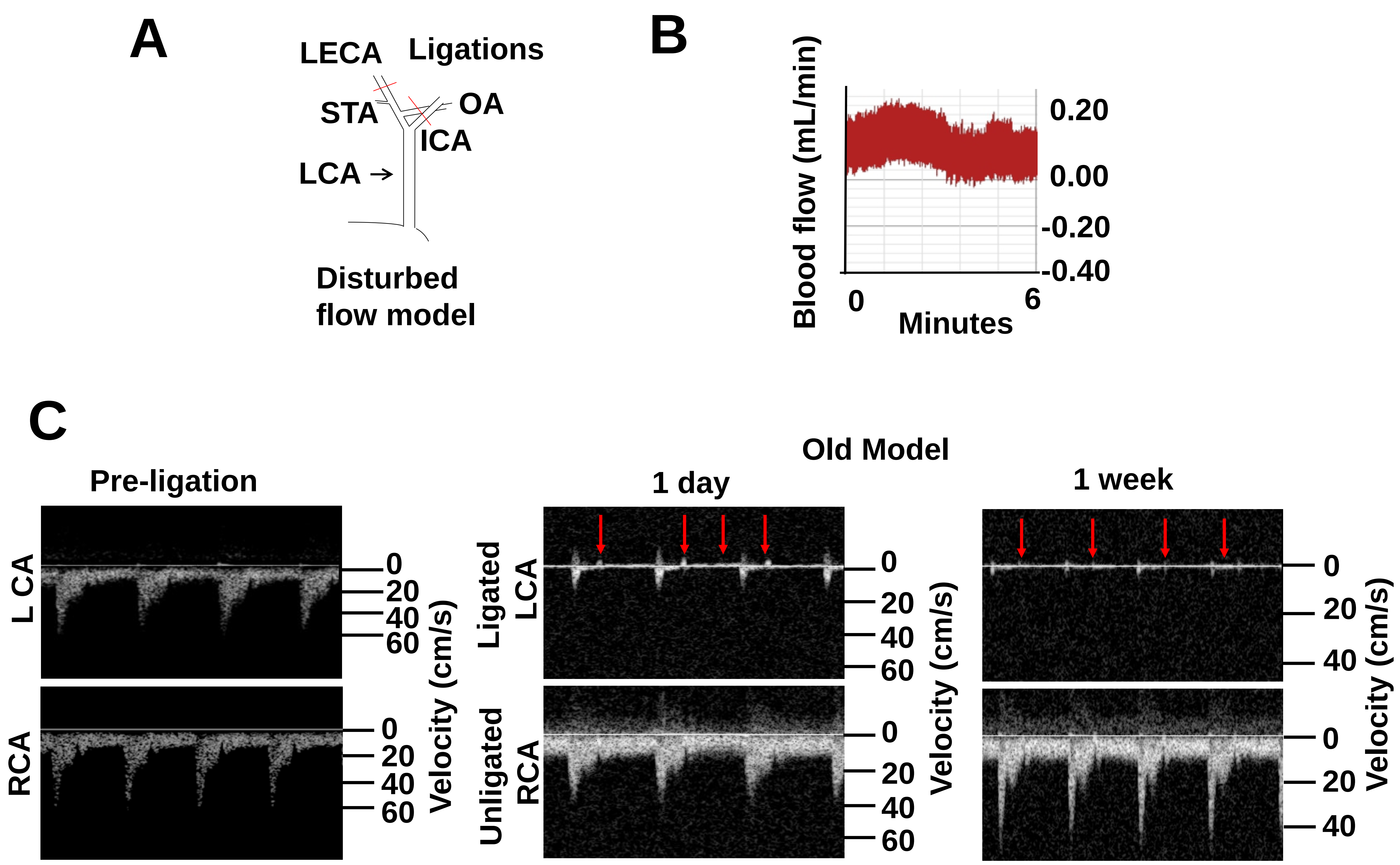

Figure S1. Measurements of blood flow in common carotid arteries in the partial ligation model.

Supplementary materials

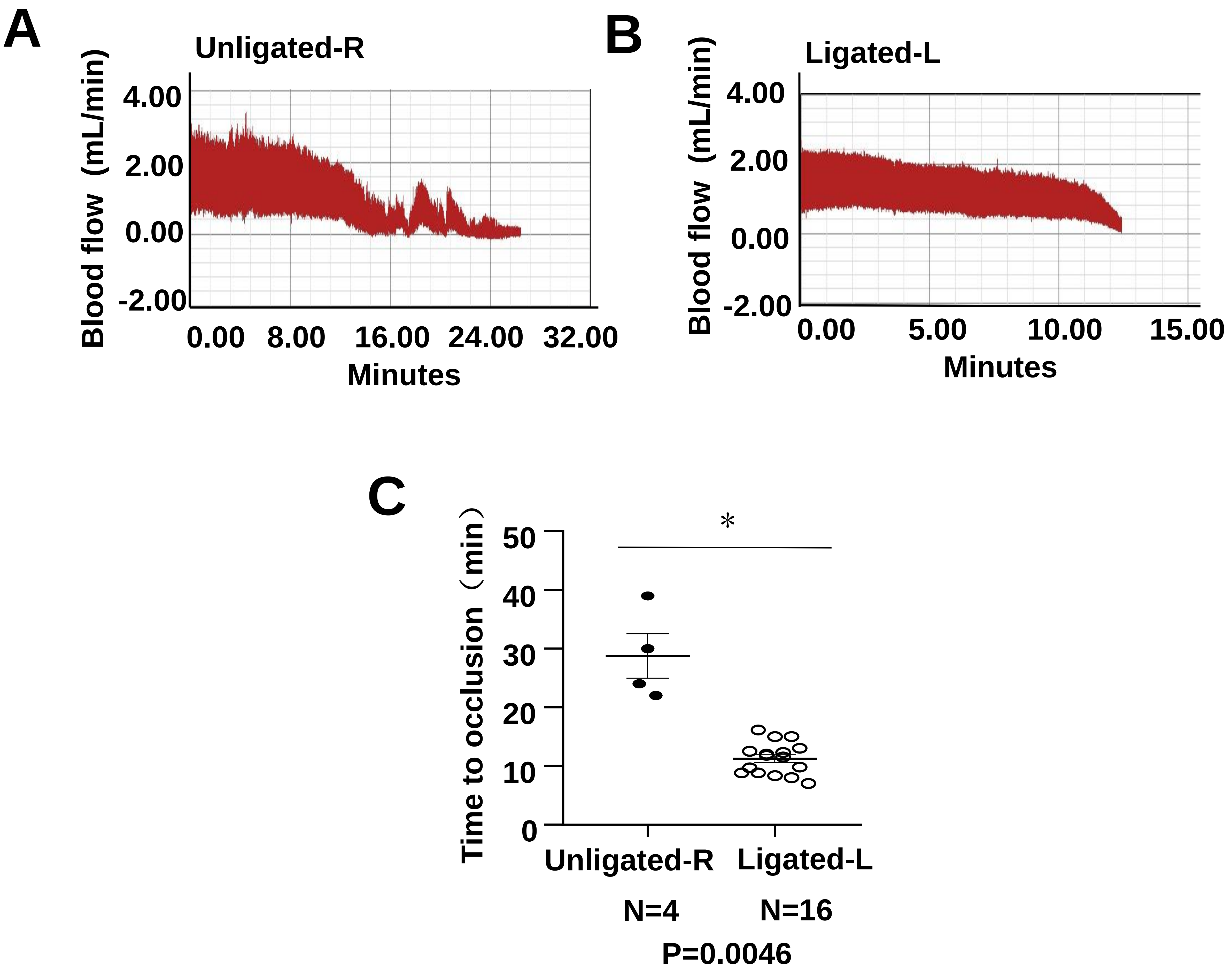

Figure S2. Ligation of LECA significantly reduces the average time to occlusion.

Supplementary materials

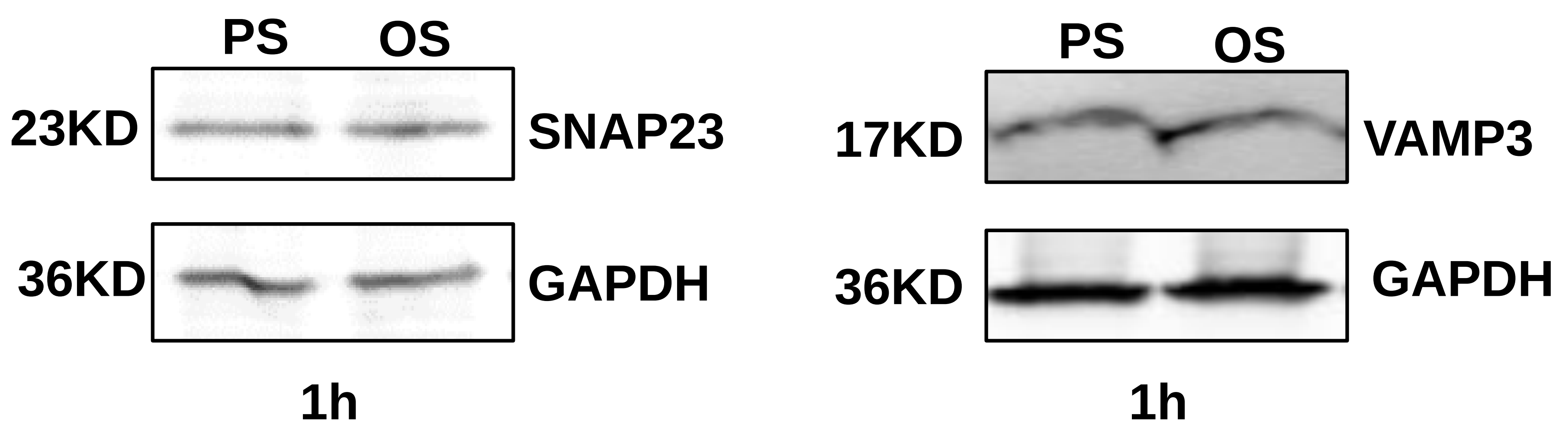

Figure S3. Influences of shear stress on the expressions of SNAP23 and VAMP3 in endothelial cells.

Supplementary materials

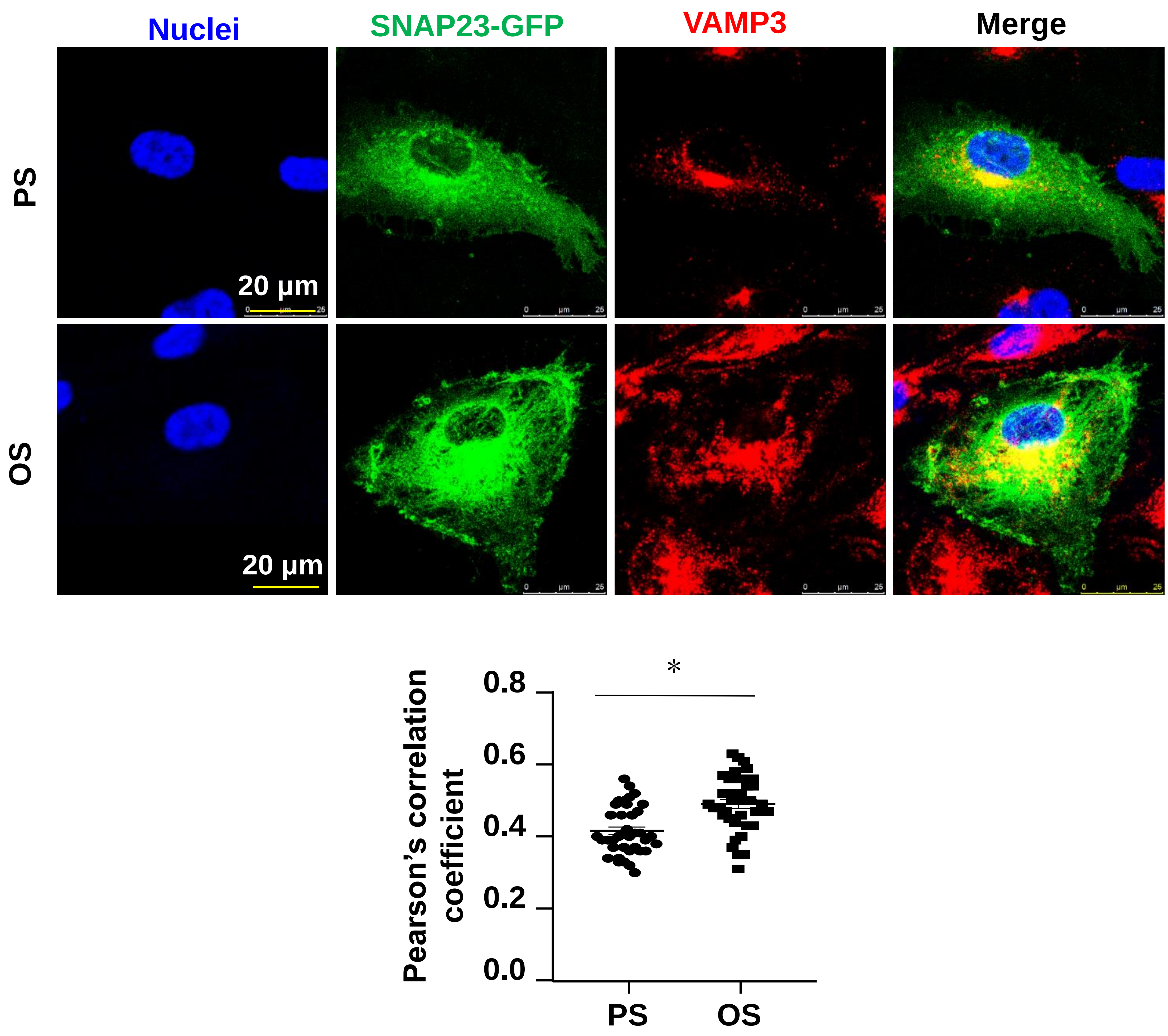

Figure S4. Oscillatory shear stress enhances the colocalization of SNAP23 and VAMP3.

Supplementary materials

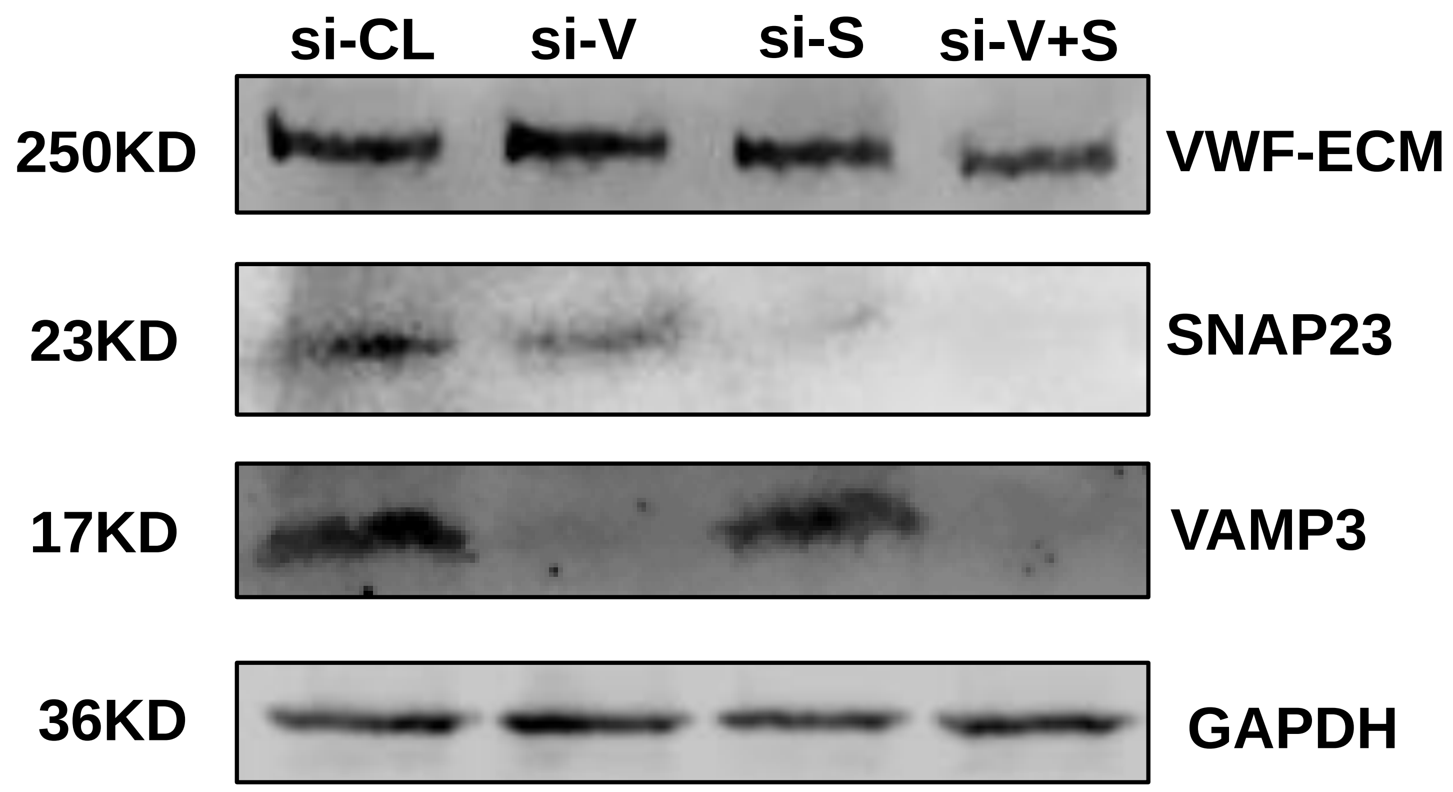

Figure S5. Knockdown of VAMP3 and SNAP23 inhibits VWF secretion.

Supplementary materials

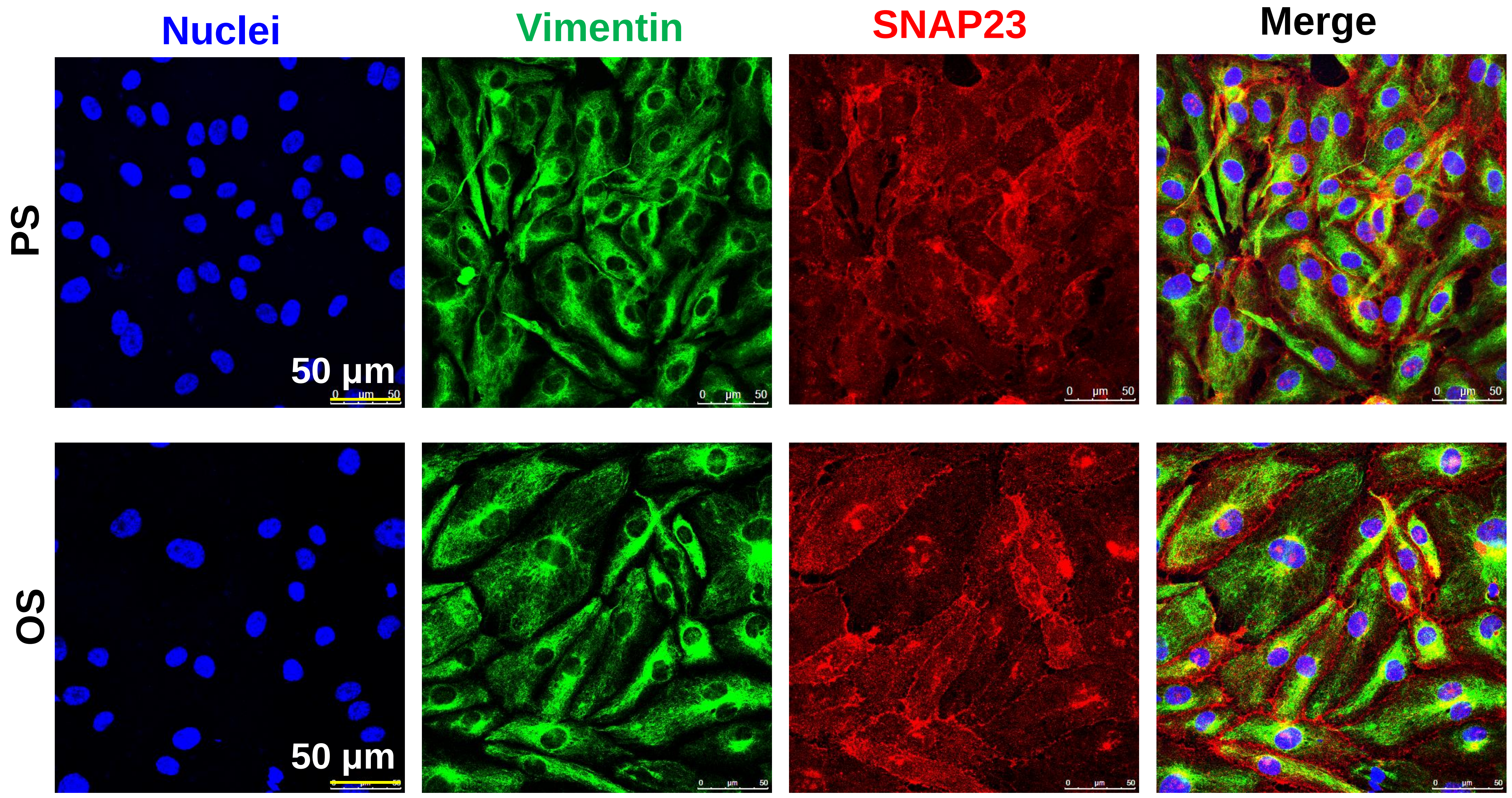

Figure S6. Effect of PS or OS on colocalization of SNAP23 and vimentin in endothelial cells.

Supplementary materials

A

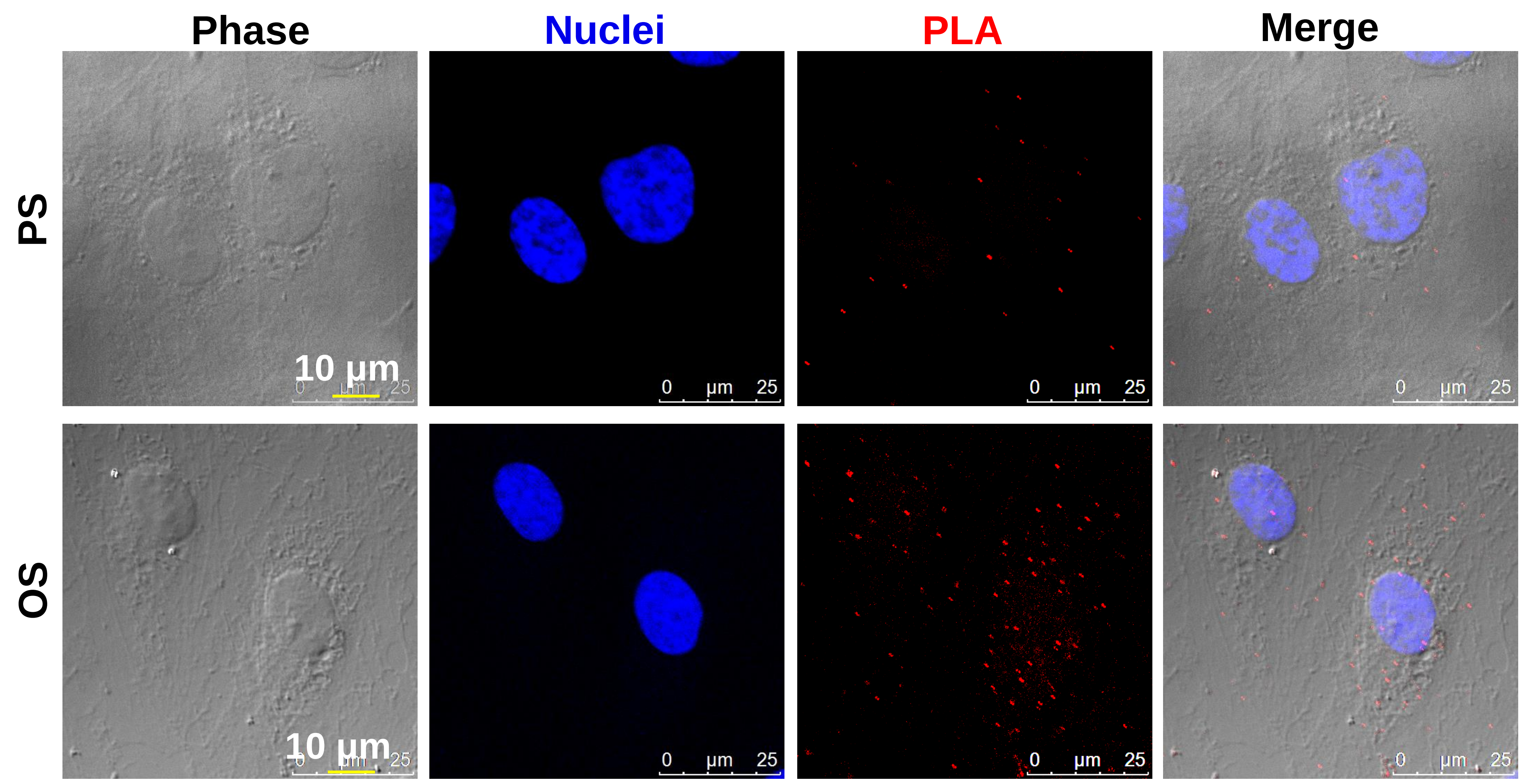

B

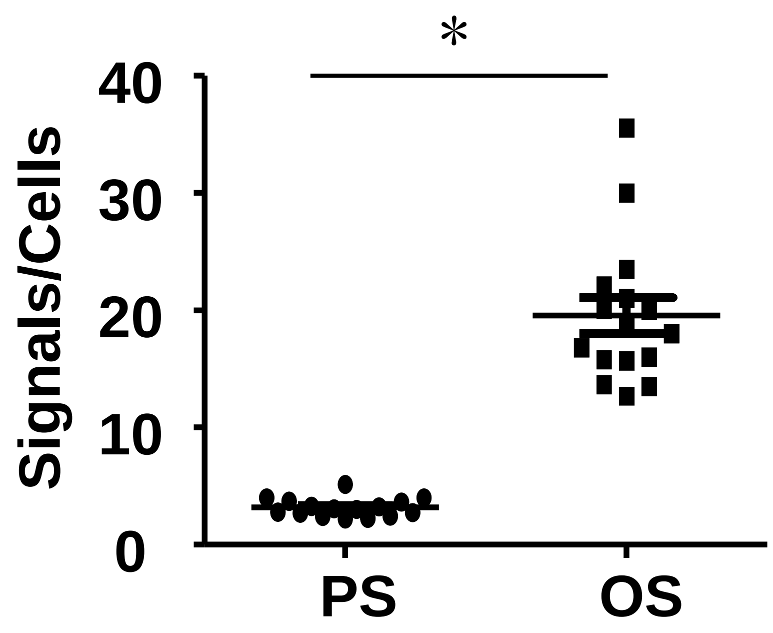

Figure S7. Oscillatory shear stress promotes the association of SNAP23 and vimentin in endothelial cells.

Supplementary materials

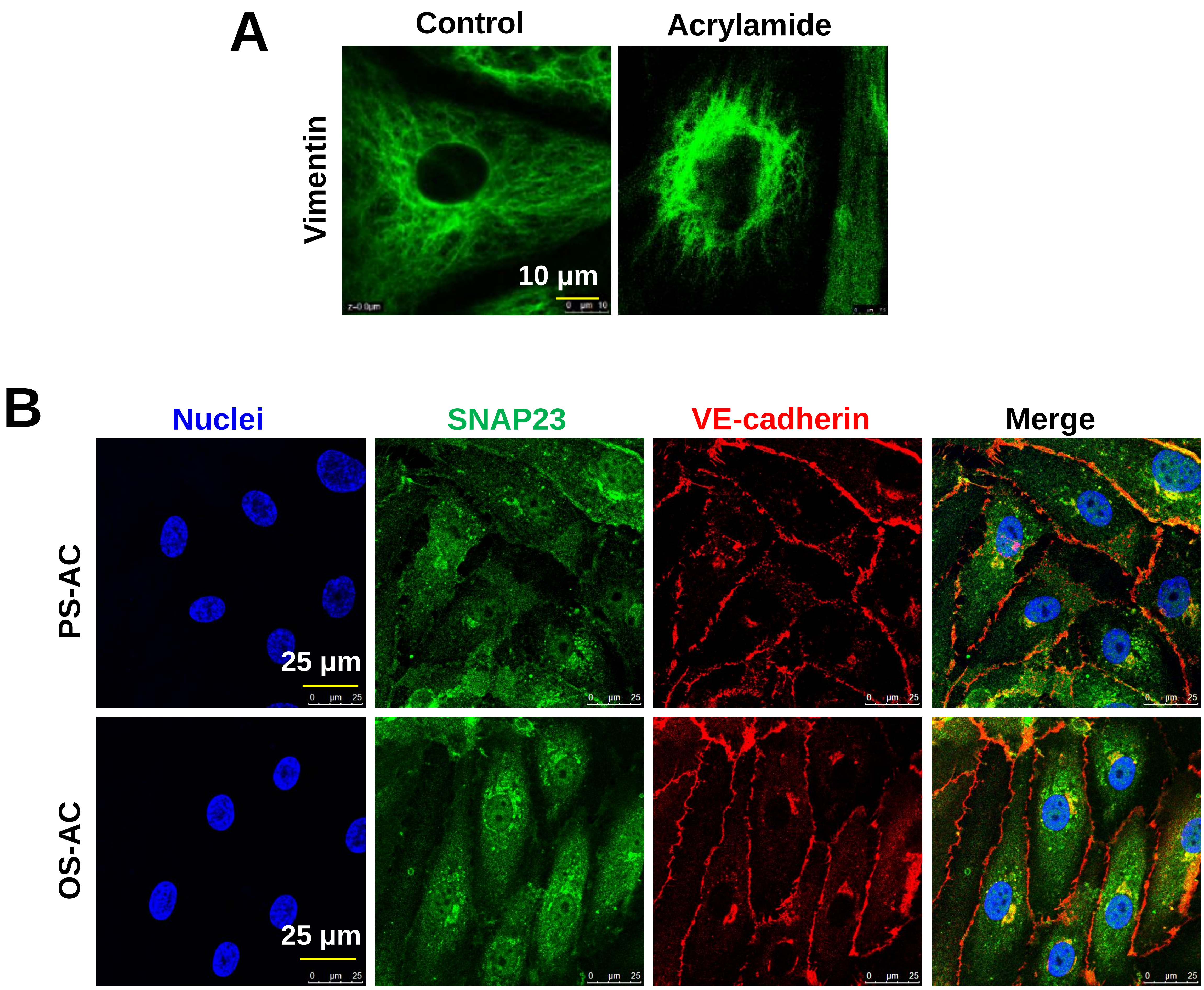

Figure S8. Treatment with acrylamide disrupts the organization of vimentin intermediate filaments as well as the cell membrane localization of SNAP23 both in static and the sheared endothelial cells.
